# Engineering 3D neural models of neurodegeneration: the Neural Microenvironment Design Code (NMDC), design principles, and emerging new approach methodologies

**DOI:** 10.1016/j.namjnl.2026.100138

**Published:** 2026-09-27

**Authors:** Alp Deniz Yuzbasic, Begum Yagmur Yesil, Damla Arslantunali Sahin, Bugra Ayan

**Affiliations:** a Department of Biotechnology, Middle East Technical University (METU), Ankara, Türkiye; b BIOMATEN, Center of Excellence in Biomaterials and Tissue Engineering, Middle East Technical University (METU), Ankara, Türkiye; c Department of Cardiothoracic Surgery, Stanford University, Stanford, CA, 94305 USA

**Keywords:** 3D bioprinting, Neural microenvironment, Neurodegenerative diseases, Biomaterials, Organoids, New Approach Methodologies (NAMs), Disease modeling

## Abstract

Neurodegenerative diseases are characterized by progressive neuronal loss driven not only by intrinsic cellular dysfunction but also by complex interactions within the neural microenvironment, including extracellular matrix (ECM) composition, neuroimmune signaling, and neurovascular dynamics. Conventional two-dimensional systems and animal models fail to recapitulate this complexity, limiting their predictive value. Recent advances in three-dimensional (3D) neural platforms, particularly 3D bioprinting and organoid technologies, have enabled the development of more physiologically relevant models that better reproduce brain architecture and multicellular organization. These systems enable the investigation of key pathological processes, including neuroinflammation, protein aggregation, and synaptic dysfunction, while aligning with emerging New Approach Methodologies (NAMs) aimed at improving human-relevant disease modeling. However, current platforms remain constrained by limited maturation, insufficient vascularization, incomplete immune integration, and experimental variability. A central premise of this review is that effective disease modeling requires not only biological complexity but also precise engineering of the neural microenvironment. To organize this engineering effort, we introduce the Neural Microenvironment Design Code (NMDC), a framework encompassing mechanical, biochemical, electrophysiological, and immune–barrier design codes that collectively define the neural microenvironment. Using the NMDC as an organizing framework, we synthesize recent advances across major neurodegenerative diseases and identify key design principles governing biomaterial selection, spatial organization, cellular integration, and biofabrication. Collectively, these approaches position 3D neural models as programmable, human-relevant systems for predictive drug screening and personalized therapeutic development.

## 1 Introduction

Three-dimensional (3D) bioprinting has emerged as a powerful platform for engineering physiologically relevant neural models, enabling precise spatial organization of cells, biomaterials, and biochemical cues (Cadena et al., 2021). This capability is particularly important in the context of neurodegenerative diseases, where disease progression is not solely driven by cellular dysfunction but by complex interactions within the surrounding microenvironment. Unlike conventional two-dimensional (2D) systems, 3D bioprinted constructs better recapitulate tissue architecture and multi-cellular organization, offering improved platforms for modeling disease-specific structure and function (Islam et al., 2024).

Neurodegenerative diseases such as Alzheimer’s disease (AD), Parkinson’s disease (PD), amyotrophic lateral sclerosis (ALS), and multiple sclerosis (MS) are increasingly recognized as disorders of the neural microenvironment, involving tightly coupled interactions between neurons, glial cells, immune components, and vascular systems. Disruption of this microenvironment, particularly through chronic neuroinflammation, extracellular matrix (ECM) remodeling, and altered cell–cell communication, plays a central role in disease progression (Bonneh-Barkay and Wiley, 2008; Stephenson et al., 2018). Therefore, effective in vitro modeling requires not only the inclusion of relevant cell types but also the ability to reconstruct the biochemical and biophysical context that governs cellular behavior. In this context, advanced 3D neural models align with emerging New Approach Methodologies (NAMs), which aim to reduce reliance on animal models while improving the predictive accuracy of human-relevant systems.

Brain tissue presents a unique challenge in this regard due to its highly specialized composition and mechanical properties. Compared to other tissues, the brain exhibits low stiffness, high water content, and a viscoelastic character, largely dictated by a non-fibrous and highly dynamic ECM. These properties, together with spatial cell organization and regional heterogeneity, create a microenvironment in which physical and biological signals are tightly interconnected (Castillo Ransanz et al., 2022). Reproducing such conditions in vitro is essential for maintaining neuronal function, synaptic activity, and disease-relevant phenotypes.

Biomaterial properties directly influence fabrication, as well as cellular behaviour, material selection is a central engineering discussion in neural bioprinting.

Advances in biofabrication strategies, including multi-material bioprinting, hybrid systems, and responsive biomaterials, have begun to address these limitations. Integration of endothelial cells (ECs), astrocytes, and pericytes into 3D bioprinted constructs enables incorporation of neurovascular features such as the blood–brain barrier (BBB), while spatially controlled architectures support the formation of functional neural networks and region- simplified models toward more predictive and physiologically relevant systems.

Despite these advances, a major challenge remains in defining how to rationally design neural microenvironments that accurately recapitulate disease-specific conditions. In this review, we present an engineering-driven perspective on 3D bioprinted neural models, focusing on the role of biomaterials, spatial organization, and microenvironmental control in neurodegenerative disease modeling. We discuss recent advances across major neurological disorders and highlight emerging design principles for constructing next-generation in vitro systems with improved physiological relevance and translational potential specific structures (Fig. 1). These developments highlight the potential of bioprinting to move beyond.

**Fig. 1:**
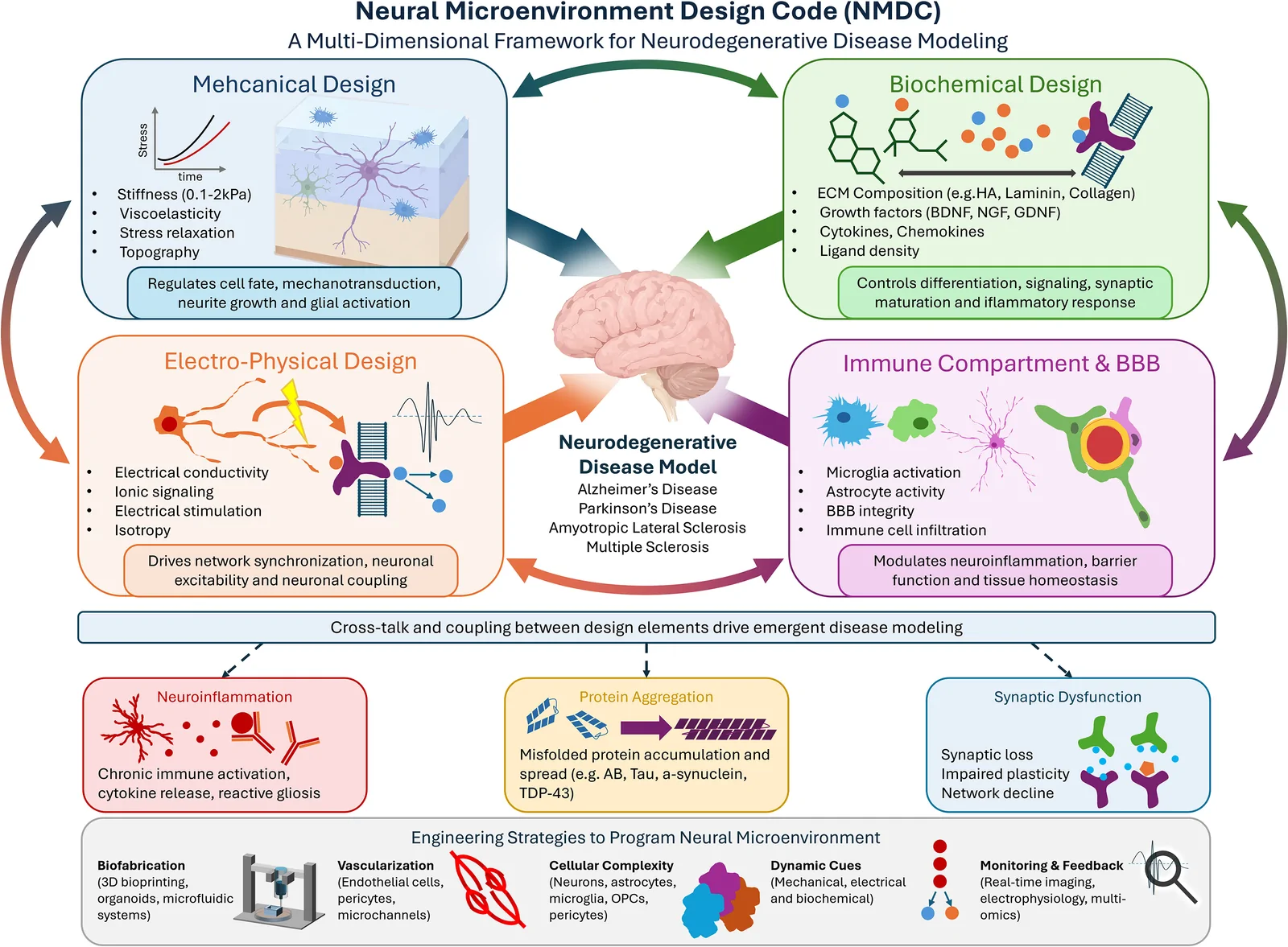
Neural Microenvironment Design Code (NMDC): A multi-dimensional framework for engineering neurodegenerative disease models.

Neurodegenerative disease phenotypes arise from the dynamic and interdependent interplay of four core microenvironmental dimensions: mechanical, biochemical, electro-physical, and immune/barrier codes. The mechanical code defines the physical properties of the matrix, including stiffness, viscoelasticity, and anisotropy, which regulate cell fate and mechanotransduction. The biochemical code encompasses ECM composition, growth factors, and cytokine signaling, governing differentiation and synaptic activity. The electro-physical code integrates electrical conductivity, ionic signaling, and fluid shear stress, enabling functional neural network behavior and neurovascular coupling. The immune and barrier code includes microglial activation, astrocyte reactivity, and BBB integrity, which collectively regulate neuroinflammation and tissue homeostasis. Importantly, these dimensions do not operate independently; instead, their interactions give rise to emergent disease phenotypes such as neuroinflammation, protein aggregation, and synaptic dysfunction. This framework provides a systems-level design strategy for developing next-generation, physiologically-relevant neural models for disease modeling and therapeutic screening.

The remainder of this review is organized in the NMDC framework. Section 3 addresses the biomaterials and biofabrication strategies exemplify the mechanical and biochemical codes; Section 4 directly proposes an engineering framework for mechanical, electrophysical, and immune/barrier codes. Subsequently, Section 5 investigates how these codes combine differently across PD, MG, ALS, AD and MS to mimic disease-specific phenotypes in vivo; and Section 6 extends the immune/barrier code to organoid systems. NMDC is intended as a guide in the context of this review, organizing each section with their respective code(s).

## 2 Properties and cells of native brain tissue

### 2.1 Cellular organization of the brain

Regional specialization within the brain is most evident in the distinct microenvironments of white and gray matter, which exhibit fundamentally different cellular architectures and physical properties (Eskandari et al., 2021). White matter primarily consists of densely packed, myelinated axonal tracts extending from neurons. Due to the parallel alignment of these axons, white matter exhibits pronounced mechanical and structural anisotropy (Feng et al., 2013). In contrast, gray matter contains a high density of neuronal cell bodies, dendrites, and complex networks of synapses between neighboring cells. Because these structures extend in multiple directions, gray matter behaves more like an isotropic material compared to white matter. Additionally, gray matter exhibits intense signaling activity, making it a highly metabolically active region with substantial baseline oxygen and glucose demands (Li et al., 2025).

The functional integrity of brain tissue is maintained by an interdependent network of specialized cellular populations. While neurons constitute the primary signaling units of the brain, other cell types—including glial and vascular cells—provide essential structural, metabolic, and protective support required for neural survival and function (Pang et al., 2026; Hernández-Peón and Sterman, 1966).

Neurons are the fundamental functional units of the central nervous system (CNS), responsible for forming complex synaptic networks that enable rapid electrical and chemical communication (Masland, 2004). This network-forming behavior makes neuronal systems highly dependent on spatial organization and connectivity, which must be presented in engineered 3D models. Among glial cell subtypes, astrocytes are the most abundant and play a central role in maintaining neural homeostasis. Through their specialized endfeet, astrocytes envelop capillaries and contribute to the maintenance of the blood–brain barrier (BBB), while also regulating local cerebral blood flow (Kugler et al., 2021; Kutuzov et al., 2018). This spatial positioning highlights the necessity of incorporating astrocytes in organized, multi-cellular systems rather than homogenous cell cultures.

Oligodendrocytes represent another essential glial population responsible for the myelination of axons within the CNS. In addition to mature oligodendrocytes, oligodendrocyte precursor cells (OPCs), also known as NG2 glia, are widely distributed throughout brain tissue and contribute to myelin remodeling and repair following injury (Zawadzka and Franklin, 2007). Myelination is strongly dependent on axonal alignment and geometry, emphasizing the importance of anisotropic architectures in engineered neural systems (Espinosa-Hoyos et al., 2018).

Other glial subtypes further support tissue homeostasis. Microglia serve as the resident immune cells of the brain, continuously surveying the microenvironment and removing cellular debris or damaged structures through phagocytic activity (Vidal-Itriago et al., 2022). Under pathological conditions such as injury or infection, microglia become activated and initiate inflammatory responses that can influence neuronal survival and synaptic remodeling (Kreutzberg, 1996).

In addition to glial cells, vascular components—ECs and pericytes—form an essential structural and regulatory network within the brain (Kugler et al., 2021; Kutuzov et al., 2018). ECs form the inner lining of cerebral blood vessels, while pericytes embedded within the vascular basement membrane regulate capillary stability, vascular permeability, and BBB integrity. Together with astrocytic endfeet and neurons, these cells form the neurovascular unit, a highly coordinated system responsible for maintaining the physiological microenvironment required for proper neural function (Muoio et al., 2014). These tightly coupled interactions impose critical design constraints on engineered systems, particularly in terms of diffusion limits, barrier function and the need for vascularization or perfusion strategies.

Collectively, these diverse cellular populations do not merely coexist within brain tissue; rather, their dynamic interactions actively regulate neural homeostasis, metabolic balance, and tissue integrity within the central nervous system. Importantly, neural tissue function arises from spatially organized and tightly coupled interactions between multiple cell types rather than from individual components. The interplay between neuronal connectivity, glial support, immune surveillance, and vascular regulation creates a complex microenvironment that is difficult to replicate in conventional in vitro systems. Therefore, effective neural tissue engineering strategies must move beyond single-cell-type approaches toward multi-cellular systems with defined spatial organization and controlled intercellular interactions.

### 2.2 Extracellular matrix composition

Compared to other tissues, brain ECM is not dominated by dense, fibrous proteins, and instead has a uniquely gel-like composition (Barros et al., 2011) and comprises approximately 20% of the total brain volume along with the brain interstitial fluid (Lei et al., 2017). This specific composition is the biological basis for the tissue's viscoelastic and highly dynamic mechanical character (Castillo Ransanz et al., 2022; Yang et al., 2021).

The structural foundation of the brain ECM is hyaluronic acid (HA), a very large unbranched glycosaminoglycan (Arranz et al., 2014). HA creates a highly hydrated biopolymer scaffold that fills the physical space between cells (Pillai and Franze, 2024). Within this hyaluronic acid scaffold are specific classes of proteoglycan that are characterized by the presence of an HA-binding domain, allowing them to anchor to the HA scaffold. Additional ECM components include chondroitin sulfate proteoglycans such as aggrecan, brevican, neurocan, and versican, which contribute to ECM structural organization and play key roles in synaptic stabilization and neuronal plasticity (Maeda, 2015). Furthermore, adhesive glycoproteins such as laminin and fibronectin are largely restricted to the vascular basal lamina rather than the interstitial space of the brain. Here, they serve as anchors between the endothelial cells and astrocyte endfeet and the surrounding matrix (Bonneh-Barkay and Wiley, 2008; Tilling et al., 2002).

Crucially, the brain ECM is not a passive scaffold, the biochemical and mechanical cues provided by the ECM regulate cell adhesion, affect cell fate decisions and migration during development, and maintain the position of cells in the adult brain (Engler et al., 2006; Franze et al., 2013). Brain ECM actively binds various growth factors and cytokines. Furthermore, synapse formation and maintenance are especially modulated with brain ECM, via the specialized ECM structures that are named perineural nets (PNNs) (Bosiacki et al., 2019).

Because the native functions and properties of the brain tissue heavily depend on this unique ECM composition, pathological conditions often involve ECM disruption (Bonneh-Barkay and Wiley, 2008). In MS, the accumulation of high-molecular-weight HA in demyelinated lesions is implicated in the physical and biochemical inhibition of oligodendrocyte maturation and remyelination (Back et al., 2005). Similarly, excessive ECM remodeling is associated with the formation of plaques and tangles in AD, altering both the biochemical and mechanical properties of the tissue (Ohkubo et al., 2003; Bruinsma et al., 2009). Importantly, the unique composition and functional role of brain ECM impose critical constraints on biomaterial design, requiring engineered systems to replicate not only its biochemical composition but also its mechanical softness, hydration and dynamic signaling capacity.

### 2.3 Mechanical properties of brain tissue

The unique, gel-like composition of the ECM, combined with densely packed cells, directly forms the basis for the biophysical and mechanical properties of native brain tissue.

The brain is one of the softest tissues in the human body and exhibits complex mechanical characteristics. Brain tissue stiffness is in the range of 0.1 to 2 kilopascals (Budday et al., 2019). The stiffness of the tissue is quantified across different spatial scales using advanced characterization techniques, such as atomic force microscopy (AFM) and indentation rheology for *in vitro* micro-scale measurements, as well as magnetic resonance elastography (MRE) for non-invasive, macro-scale mapping *in vivo* (Morr et al., 2022; Cieśluk et al., 2020). Although this range is relatively narrow, the brain exhibits significant spatial heterogeneity in stiffness (Bouchonville et al., 2016). As mentioned in the previous section, the cellular architecture of gray and white matter differs significantly, and this difference also accounts for variations in stiffness (Budday et al., 2015). Gray matter is composed primarily of neuronal cell bodies, dendrites, and unmyelinated axons, and it generally behaves as an isotropic material (Feng et al., 2013). Its stiffness and deformation remain relatively similar regardless of the direction of the applied mechanical stress. White matter, on the other hand, is composed of tightly packed myelinated axonal tracts and is highly anisotropic (Eskandari et al., 2021). It is significantly stiffer when stretched or compressed along the direction of axonal bundles compared to when the force is applied in a different direction (Budday et al., 2015).

Another important mechanical property of brain tissue is viscoelasticity (Miller et al., 2000). Thus, the biological response to mechanical forces is time- and rate-dependent. The viscoelasticity of the brain is partially the result of friction between HA polymers, rearrangement of the cytoskeleton, and the sliding motion of cell membranes against one another. When subjected to a sudden impact, such as traumatic brain injury, the viscous components of the tissue restrict rapid deformation, causing the brain to behave like a stiffer, solid-like material (Hosseini-Farid et al., 2020). Conversely, under slow and sustained forces, such as the expansion of a tumour, the tissue adapts to the applied force and behaves in a more fluid-like manner (Streitberger et al., 2020). This strain-rate dependence allows the brain to absorb and dissipate small-scale mechanical forces while remaining responsive to cellular movements and plasticity.

A critical feature of the brain's viscoelastic character is stress relaxation. This relaxation behaviour occurs because the cytoskeleton of cells, ECM components, and cell membranes dynamically reorganize and dissipate stored mechanical energy. At the cellular level, stress relaxation is an important mechanical cue (Qiao et al., 2024). When cells extend processes and pull on their microenvironment, they do not experience a static rigid resistance; instead, they sense these time-dependent mechanical changes through mechanotransduction pathways, where mechanical cues from the environment are transformed to biochemical signals (Qiao et al., 2024; Saraswathibhatla et al., 2023; Stukel and Willits, 2016). The specific rate at which brain tissue relaxes directly affects how CNS cells migrate, form synapses, and maintain homeostasis (Castillo Ransanz et al., 2022; Roth et al., 2023).

Brain tissue exhibits poroelastic behaviour, in which mechanical responses arise from the coupled interaction between a deformable solid and interstitial fluid flow within the tissue (Su et al., 2023).

When a mechanical force is applied to the brain, part of the mechanical response arises from the movement of the fluid phase. This behavior is largely due to the narrow pores present in brain tissue; the interstitial fluid encounters significant frictional resistance when forced to move through the matrix. Therefore, while the viscoelastic character of the brain originates from molecular friction and structural rearrangement within the solid phase, the poroelastic character stems from the displacement of the fluid phase moving through the dense matrix (Su et al., 2023; Budday et al., 2017). Together, these properties give rise to the brain tissue’s ability to distribute mechanical pressure across the organ, effectively acting as a shock absorber.

Collectively, the extreme softness, viscoelasticity, stress relaxation, and poroelasticity of brain tissue create a highly dynamic biophysical environment. Cells within brain tissue, as described in the previous sections, continuously probe these mechanical properties to adjust their function and are finely tuned to this specific mechanical environment. They sense the stiffness and relaxation rates of their surroundings to guide development and function. Consequently, deviations from native tissue properties act as pathological signals that disrupt cellular homeostasis. During aging and in neurodegenerative diseases such as AD, PD, and MS, the mechanical properties of brain tissue become altered, often showing softening associated with neuronal loss and inflammation (Wuerfel et al., 2010; Arani et al., 2015; Murphy et al., 2011). Although broad-level softening is observed in these diseases, each condition is accompanied by distinct mechanical deviations. For example, in AD, tissue exhibits pronounced mechanical heterogeneity due to rigid plaque formation accompanying overall softening (Murphy et al., 2015).

### 2.4 Neuroimmune interactions and neuroinflammation

Historically, the nervous and immune systems were viewed as functionally isolated, communicating primarily through long-range neuroendocrine signaling (Guillemin, 1978). However, studies of both systems have revealed a highly integrated network of local and short-range interactions. These communication pathways, directed both from neuronal components to immune components and vice versa, are essential for normal CNS development and homeostasis (Tian et al., 2012; Dantzer, 2017). When dysregulated, however, neuroinflammation becomes a major driver of CNS pathology and neurodegeneration. The core components of this neuroimmune communication include microglial activation, cytokine signaling, and immune cell infiltration (Balistreri and Monastero, 2023).

Microglia, the resident macrophages of the CNS, function as a type of surveillance system in the healthy brain. Under conditions of stress or injury, microglia become activated and shift their role from supportive surveillance to potentially neurotoxic and pro-inflammatory functions. During this activation state, they downregulate homeostatic activities that support synaptic plasticity and neuronal survival while releasing inflammatory mediators and reactive oxygen species (Guo et al., 2022). Working in collaboration with microglia, astrocytes are also the central players in neuroinflammation. In response to injury or cross talk with microglia, astrocytes undergo reactive astrogliosis (Escartin et al., 2021).

Another important component of the neuroimmune environment is cytokine signaling (Kennedy and Silver, 2022). Cytokines are signaling proteins secreted by cells within brain tissue and function as the molecular language between the immune and nervous systems (Dantzer, 2017). They also act as neuromodulators in the healthy brain. Under physiological conditions, cytokines are constitutively expressed and contribute to the regulation of synaptic strength and plasticity (Zipp et al., 2023).

Finally, although the CNS is protected by the BBB, severe or chronic neuroinflammation can compromise this barrier and allow peripheral immune cells to infiltrate the brain parenchyma (Zlokovic, 2008; Louveau et al., 2015). This prolonged neuroinflammatory state can create highly toxic microenvironments and further drive the progression of neurodegenerative processes (Netzahualcoyotzi et al., 2024). Importantly, neuroimmune interactions are highly dynamic and context-dependent, making them difficult to replicate in simplified in vitro systems. Effective neural models must therefore, incorporate immune components and enable controlled modulation of inflammatory signals to capture disease relevant microenvironments.

### 2.5 Neurovascular interactions

The metabolic demands of the brain are exceptionally high relative to its size. To support this intense metabolic activity, cerebral blood flow and barrier functions are tightly regulated by a coordinated cellular network known as the neurovascular unit (NVU). The NVU comprises ECs, mural cells—including pericytes in capillaries and vascular smooth muscle cells in arteries—the vascular basal lamina, astrocytic endfeet, and local interneurons (Sweeney et al., 2018). Together, these components form an integrated regulatory system that maintains the physiological microenvironment required for proper neural function.

In the adult brain, the NVU plays a central role in regulating the transport of substances between the systemic circulation and the brain parenchyma. Cerebral ECs establish the BBB by forming tight junctions that restrict paracellular transport (Yu et al., 2015). In addition to its structural tight junctions, the BBB also relies on highly selective transport systems that regulate the controlled exchange of nutrients, metabolites, and signaling molecules (Yu et al., 2015).

Importantly, the specialized properties of the BBB are not determined by endothelial cells alone. Continuous signaling interactions between ECs, pericytes, and astrocytic endfeet are essential for the induction and maintenance of tight junction integrity and barrier selectivity (Zlokovic, 2008; Abbott et al., 2010). Pericytes play a critical role in regulating vascular stability, permeability, and capillary blood flow, thereby contributing significantly to the functional integrity of the BBB (Daneman et al., 2010).

Neural activity and cerebral blood flow are closely linked through a process known as neurovascular coupling. This mechanism ensures that metabolically active brain regions receive increased blood supply, thereby matching local energy demand with oxygen and glucose delivery (Cai et al., 2018).

In addition to regulating blood flow, the neurovascular unit contributes to metabolic waste clearance through its interaction with the glymphatic system, a brain-wide fluid transport pathway that facilitates the removal of metabolic by-products such as β-amyloid (Hablitz and Nedergaard, 2021).

Neurovascular dysfunction such as reduced cerebral blood flow, disruption of the BBB, and impaired clearance of metabolic waste are increasingly recognized as key contributors to neurodegenerative diseases (Dagum et al., 2026; Chen et al., 2024; Wang et al., 2025).

## 3 Biomaterials and biofabrication strategies for neural 3D bioprinting

Within the NMDC framework, this section addresses biomaterials and biofabrication strategies that realize the mechanical and biochemical codes; which translates the neural microenvironment design targets to bioprintable and material systems. Biomaterials play a central role in neural 3D bioprinting, as they define not only the bioprintability and structural stability of engineered constructs but also the biological microenvironment that regulates cellular behavior. In neural tissue engineering, this relationship is particularly critical because neurons, astrocytes, oligodendrocytes, and neural stem/progenitor cells are highly sensitive to the physicochemical properties of the surrounding matrix. Parameters such as stiffness, viscoelasticity, porosity, degradation profile, water content, and biochemical ligand presentation collectively influence cell survival, migration, differentiation, and network maturation (Tsou et al., 2016). Thus, biomaterials function as active regulators of both cellular behaviour and tissue-level organization.

To systematically address these competing demands, neural bioink design can be conceptualized within three interconnected dimensions: (i) biological functionality, (ii) mechanical and structural fidelity, and (iii) manufacturability through bioprinting. These dimensions are inherently interdependent, and successful neural bioinks must balance them to achieve both physiological relevance and fabrication feasibility (Khan and Ahmed, 2026; Theus et al., 2020). Importantly, optimization along a single dimension is insufficient, as improvements in one parameter often compromise others.

This framework highlights a fundamental challenge in neural bioprinting: the need to reconcile biological relevance with structural and manufacturing constraints. In this context, biomaterial selection is no longer based solely on cytocompatibility or ease of processing, but on the ability to balance multiple, often competing, demands including bioprintability, matrix compliance, degradation behavior, and bioactivity (Zhang et al., 2021; Gungor-Ozkerim et al., 2018). This shift reflects a transition from material selection to material system design.

The neural ECM is not a passive scaffold; rather, it is a dynamic and instructive environment that regulates tissue organization, synaptic stabilization, and cell signaling (Rauti et al., 2020). This is especially relevant for disease modeling, where subtle differences in matrix properties may influence cellular phenotypes and alter the physiological relevance of the model. Consequently, even minor deviations from native ECM properties can lead to significant changes in model outcomes.

Overall, biomaterials for neural 3D bioprinting must be understood as active regulators of both biofabrication success and biological outcome. This requires integrated design strategies that simultaneously consider material properties, bioprinting constraints, and cell-specific responses across multiple spatial and temporal scales.

### 3.1 Design considerations for neural bioinks

From an NMDC perspective, neural bioink design implements the mechanical and biochemical codes simultaneously, since stiffness, ligand presence and matrix properties must be optimized in parallel within a single system. The design of neural bioinks requires the careful integration of mechanical, biological, and processing-related parameters. In contrast to conventional scaffold design, neural bioinks must simultaneously support highly sensitive cell populations while enabling the reliable fabrication of structurally stable and spatially defined 3D constructs. As a result, neural bioink development is best understood as a materials design problem defined by trade-offs between cytocompatibility, matrix fidelity, and bioprintability (Choi et al., 2025; Knowlton et al., 2018). These competing requirements necessitate a multi-parameter design strategy where biological performance, mechanical behavior and processing constraints must be optimized simultaneously rather than sequentially. Importantly, optimization within a single parameter space is insufficient to achieve functional neural constructs.

Mechanical compliance represents one of the most critical parameters in neural tissue engineering. Neural tissues are among the softest in the human body, and even modest deviations from physiological stiffness can significantly alter cellular behavior. Therefore, neural bioinks must be carefully tuned to approximate native tissue mechanics while remaining compatible with the constraints of the selected bioprinting modality (Deng et al., 2025; Wu et al., 2019). This requirement establishes stiffness as a primary design variable rather than a secondary material property.

Microstructural properties, including porosity and network architecture, further influence the performance of neural bioinks. At the same time, matrix architecture determines the ability of cells to migrate, extend neurites, and form interconnected networks. This balance is strongly influenced by polymer concentration, crosslinking density, and degradation behavior (Peppas et al., 2006). Thus, microstructure must be engineered to enable both cell motility and structural integrity.

Biochemical functionality is another key determinant of bioink performance. Neural cells rely heavily on extracellular cues for maturation, organization, and synaptic formation. Natural biomaterials often contain intrinsic adhesion motifs and cell-instructive domains, whereas synthetic materials typically require functionalization to achieve comparable bioactivity. Incorporation of ECM-derived components such as laminin, collagen-derived peptides, or HA-based molecules can significantly enhance neuronal attachment, neurite guidance, and lineage-specific differentiation. These cues are essential for reproducing physiologically relevant neural phenotypes in vitro (Kratochvil et al., 2019; Ji and Guvendiren, 2017).

Crosslinking strategy plays a central role in determining both mechanical stability and cytocompatibility. Stronger crosslinking generally improves shape retention and long-term structural integrity but may also expose cells to harmful conditions such as reactive radicals, ultraviolet irradiation, or cytotoxic chemical agents. Given the sensitivity of neural cells, mild crosslinking approaches including ionic gelation, enzymatic crosslinking, and thermally induced gelation are often preferred. The choice of crosslinking chemistry must therefore balance fabrication requirements with the preservation of cellular function over both short and long-time scales (Sharifi et al., 2021; Ulubayram et al., 2002; Wang et al., 2013). This trade-off highlights crosslinking as a critical control point in bioink design.

Bioprintability introduces an additional layer of complexity, as the rheological properties of the bioink directly influence deposition fidelity and cell viability. However, increasing viscosity to improve bioprintability often results in elevated shear stress during extrusion, which can reduce cell viability, particularly for neural cells that are highly sensitive to mechanical damage. To address this challenge, current strategies frequently employ shear-thinning hydrogels, sacrificial support systems, and embedded bioprinting approaches that enable the use of softer, more cell-friendly bioinks without compromising structural resolution (Sánchez-Sánchez et al., 2023; Chen et al., 2017). These strategies effectively decouple bioprintability from biological permissiveness.

An additional and increasingly important consideration is the spatiotemporal complexity of neural tissue. Static and compositionally uniform hydrogels often fail to capture this complexity. Consequently, there is growing interest in gradient bioinks, multi-material systems, stimuli-responsive matrices, and dynamically remodeling hydrogels that can reproduce both spatial and temporal variations in the cellular microenvironment (Pei et al., 2021; Bracaglia et al., 2017). This shift reflects a transition toward dynamic and programmable biomaterial systems.

### 3.2 Natural biomaterials for neural 3D bioprinting

Natural biomaterials remain central to neural 3D bioprinting because of their intrinsic bioactivity, high water content, and biochemical similarity to the native ECM. Unlike many synthetic polymers, naturally derived hydrogels inherently provide cell-adhesive ligands, matrix remodeling sites, and biochemical signals that support neural cell survival, neurite extension, and synaptic maturation (Bupphathong et al., 2022; Amirrah et al., 2022; Buckley et al., 2022). These features are particularly important in neural tissue engineering, where cellular behavior is highly dependent on subtle microenvironmental cues.

A major advantage of natural biomaterials is their ability to recreate the soft and hydrated characteristics of brain tissue. Materials such as gelatin, collagen, fibrin, and HA exhibit mechanical properties within or near the low kilopascal range of native neural tissue, thereby promoting neuronal differentiation, network formation, and glial homeostasis (Budday et al., 2019; Buckley et al., 2022; López-León et al., 2024). In addition, ECM-derived components including laminin and brain-derived decellularized extracellular matrix (dECM) provide tissue-specific biochemical signals that enhance physiological relevance and improve neural maturation (Calado et al., 2026; Kort-Mascort et al., 2023).

Despite these advantages, natural biomaterials also present significant engineering limitations. Most exhibit weak mechanical stability, rapid degradation, and poor print fidelity when used alone, making it difficult to maintain complex 3D architectures during and after bioprinting (Wan et al., 2024; Asim et al., 2023; Sanz-Horta et al., 2023). Furthermore, several biologically active matrices, including Matrigel and dECM, suffer from batch-to-batch variability and limited standardization, which complicate reproducibility and translational scalability (Calado et al., 2026; Seok et al., 2024). Consequently, the primary challenge in neural bioprinting is not simply identifying biologically compatible materials but engineering natural biomaterial systems that preserve bioactivity while enabling controlled fabrication.

To address these limitations, current strategies increasingly rely on chemical modification and composite bioink design. Gelatin is frequently converted into gelatin methacrylate (GelMA) to improve structural stability through photocrosslinking, while HA is modified into photocrosslinkable derivatives such as HAMA to enhance mechanical tunability and bioprintability (Sharifi et al., 2021; Ghosh et al., 2023; Falcone et al., 2006). Similarly, biologically permissive matrices such as fibrin, laminin, and dECM are commonly integrated with mechanically supportive polymers including alginate, GelMA, or synthetic hydrogels to balance cytocompatibility with structural integrity (Kort-Mascort et al., 2023; Sanz-Horta et al., 2023).

Importantly, natural biomaterials should not be viewed solely as passive scaffolds. Rather, they function as instructive microenvironments that actively regulate neural differentiation, neuroimmune interactions, synaptic organization, and tissue-level maturation. As a result, their role in neural bioprinting extends beyond simple cell encapsulation toward the reconstruction of dynamic and physiologically-relevant neural micro-environments (Choi et al., 2025).

#### 3.2.1 Bioactive structural matrices: Gelatin, collagen, and fibrin

Gelatin, collagen, and fibrin are among the most widely used natural biomaterials in neural tissue engineering due to their strong bioactivity and ECM-mimetic characteristics. These materials support neural cell adhesion, migration, and neurite extension through intrinsic biochemical motifs and favorable hydration properties (Bupphathong et al., 2022; Amirrah et al., 2022; Yu et al., 2020). Gelatin contains arginine–glycine–aspartic acid (RGD) motifs that facilitate neuronal attachment and differentiation, while collagen provides fibrillar structural cues that regulate integrin-mediated cell interactions (Bupphathong et al., 2022; Lee et al., 2011). Fibrin further contributes through its highly cell-permissive architecture and physiological gelation mechanism, which enable cytocompatible encapsulation under mild conditions (Yu et al., 2020).

Despite their biological advantages, these materials exhibit limited mechanical robustness and poor long-term structural stability when used as standalone bioinks (López-León et al., 2024; Asim et al., 2023; Sanz-Horta et al., 2023). Gelatin undergoes thermally induced softening near physiological temperatures, collagen hydrogels are prone to rapid degradation and contraction, and fibrin rapidly degrades through enzymatic remodeling (López-León et al., 2024; Asim et al., 2023). Consequently, these systems are frequently modified or incorporated into hybrid bioinks to improve bioprintability and structural fidelity. Common approaches include methacrylation of gelatin and collagen, interpenetrating polymer networks, and blending with mechanically supportive polymers such as alginate or PEG-based hydrogels (Sharifi et al., 2021; Islam et al., 2021; Yue et al., 2025). Consequently Gelatin, GelMA, Fibrin based hydrogels and bioinks are frequently employed in low concentrations, usually below 10% (w/v) mimicking physical properties of neural tissue compartments. Models of diseases with pronounced neuroinflammation or connective tissue interactions, such as AD and MG would benefit greatly from accurate mechanical and biochemical design through mentioned biomaterials. Thus, in context of NMDC, while these materials may be sufficient per mechanical design considerations, immune design and biochemical design aspects must also be considered, since such materials present bioactive sites and even possible xenogenic motifs that may induce immune response and neruoinflammation depending on the source and other biomaterials used to formulate hybrid bioinks.

#### 3.2.2 Hyaluronic acid-based bioinks

HA occupies a unique position in neural tissue engineering because it is one of the principal glycosaminoglycans of the native brain ECM and plays a central role in regulating tissue hydration, viscoelasticity, and cell signaling (Buckley et al., 2022). Unlike fibrillar ECM proteins that dominate many peripheral tissues, HA-rich matrices contribute to the highly hydrated and mechanically compliant nature of neural tissue (Arranz et al., 2014; Pillai and Franze, 2024). In addition to its structural role, HA interacts with receptors such as CD44 and RHAMM, influencing neural stem cell behavior, migration, and differentiation (Ngo et al., 2020; Ma et al., 2023).

Importantly, the biological activity of HA is strongly dependent on molecular weight and matrix organization. High molecular weight HA is generally associated with anti-inflammatory and neuroprotective effects, whereas fragmented or low molecular weight HA can promote inflammatory signaling and tissue remodeling (Ngo et al., 2020; Pérez-Lloret and Erxleben, 2024; Misra et al., 2015). This dynamic behavior makes HA-based systems particularly attractive for modeling neuroinflammation and disease-associated ECM remodeling.

However, native HA hydrogels typically lack sufficient mechanical integrity and print fidelity for standalone bioprinting applications (Falcone et al., 2006). To overcome these limitations, HA is commonly modified into photocrosslinkable derivatives such as hyaluronic acid methacrylate (HAMA) or incorporated into composite systems with GelMA, alginate, or synthetic polymers (Falcone et al., 2006). These approaches enable independent tuning of stiffness, degradation, and rheological behavior while preserving the biological relevance of HA-rich microenvironments.

#### 3.2.3 Structural support bioinks: Alginate-based systems

Alginate is widely used in neural bioprinting primarily because of its favorable rheological properties and rapid ionic crosslinking behavior (Sahoo and Biswal, 2021; Wang et al., 2023). Through calcium-mediated gelation, alginate enables reproducible fabrication of structurally stable hydrogel architectures with high shape fidelity, making it particularly suitable for extrusion-based bioprinting (EBB) approaches (Liu et al., 2022). In this context, alginate serves primarily as a structural support component that enhances printability and mechanical stability.

Unlike many ECM-derived biomaterials, however, alginate is biologically inert and lacks intrinsic cell-adhesive ligands (Sahoo and Biswal, 2021). As a result, alginate alone generally provides insufficient support for neural attachment, neurite extension, and long-term functional maturation. To address this limitation, alginate is frequently functionalized with bioactive peptides such as RGD or blended with neural ECM components including gelatin, laminin, collagen, or HA (Liu et al., 2022). However, absence of bioactive sites imparts a certain amount of stealth behavior to high purity alginate-based constructs which may be beneficial. It has been shown that pure alginate constructs do not cause acute foreign body reactions by the immune system in CNS implants. In context of NMDC, properties of alginate-based bioinks can be exploited in neural disease model design for its low bioreactivity during biochemical design and for its low immunogenicity in immune compartment design. (El-Sheekh et al., 2026; Farshidfar et al., 2023) These hybrid formulations combine the favorable processing characteristics of alginate with the biological functionality required for neural tissue formation.

#### 3.2.4 ECM-derived bioinstructional systems: Laminin, matrigel, and dECM

ECM-derived biomaterials such as laminin, Matrigel, and brain-derived decellularized Extracellular Matrix (dECM) provide some of the most biologically relevant microenvironments currently available for neural tissue engineering (Kort-Mascort et al., 2023; Rogers and Nishimune, 2017). These systems contain complex combinations of ECM proteins, glycosaminoglycans, and signaling molecules that regulate neuronal differentiation, neurite guidance, synaptic maturation, and tissue organization (Calado et al., 2026; Zhang et al., 2026). As a result, ECM-derived matrices are widely used to enhance the physiological relevance of neural organoids, assembloids, and bioprinted neural constructs.

Among these materials, laminin plays a particularly important role in neural development and basement membrane organization, while Matrigel provides a highly permissive environment for neural differentiation due to its rich composition of laminin, collagen IV, and growth factors (Calado et al., 2026; Rogers and Nishimune, 2017; Zhang et al., 2026; Jury et al., 2022; Ozbek et al., 2024). Similarly, brain-derived dECM retains tissue-specific biochemical cues that are difficult to reproduce using simplified synthetic or single-component biomaterials, thereby improving neural maturation and disease-specific phenotypes (Kort-Mascort et al., 2023; Abaci and Guvendiren, 2020; Saldin et al., 2016; Yeleswarapu et al., 2023; Faghih et al., 2025; Zhang et al., 2021).

Nevertheless, these systems also present substantial translational limitations. Matrigel exhibits significant batch variability and xenogenic origin, whereas dECM-based materials often suffer from inconsistent composition, limited scalability, and poor mechanical stability (Calado et al., 2026; Seok et al., 2024).

### 3.3 Synthetic biomaterials for neural 3D bioprinting

Synthetic biomaterials play a critical role in neural 3D bioprinting by providing precise control over mechanical properties, degradation behavior, and biofabrication performance; which makes them mainly related to mechanical code in NMDC terms . In contrast to natural biomaterials, whose biological and mechanical properties are often intrinsically coupled, synthetic polymers enable independent tuning of stiffness, porosity, crosslinking density, and rheological behavior (Mahoney and Anseth, 2006; Papadimitriou et al., 2020). This tunability is particularly important in neural tissue engineering, where subtle variations in matrix mechanics and architecture can significantly influence neuronal differentiation, glial activation, and network formation.

However, the primary limitation of most synthetic biomaterials is their intrinsic bioinertness. Unlike ECM-derived matrices, synthetic polymers generally lack cell-adhesive ligands and tissue-specific biochemical signals, limiting their ability to support neural attachment, neurite extension, and functional maturation in their native form (Parak et al., 2019; Khoeini et al., 2021; Kumar, 2021). Consequently, synthetic systems frequently require biochemical functionalization or integration with natural biomaterials to achieve physiologically relevant neural microenvironments.

Rather than serving as direct replacements for natural ECM, synthetic biomaterials are increasingly utilized as tunable engineering platforms that complement biologically active matrices. Through chemical modification, incorporation of bioactive peptides, and hybrid bioink design, synthetic systems enable decoupling of structural and biological functions, allowing independent optimization of fabrication and cellular performance (Doblado et al., 2021). This transition from standalone materials toward modular and multifunctional biomaterial systems represents a major conceptual shift in neural biofabrication.

#### 3 3.1. Tunable hydrogel platforms

Polyethylene glycol (PEG)-based hydrogels are among the most extensively studied synthetic systems in neural tissue engineering due to their hydrophilicity, biocompatibility, and highly tunable network properties (Xu et al., 2018). Photocrosslinkable derivatives such as PEG diacrylate (PEGDA) and PEG methacrylate (PEGMA) allow precise regulation of stiffness, porosity, degradation, and crosslinking density, enabling the fabrication of structurally defined neural constructs with high reproducibility (Yue et al., 2025).

One of the defining features of PEG is its resistance to nonspecific protein adsorption and uncontrolled cellular interactions (Lee et al., 2016). While this characteristic improves experimental reproducibility, it also limits neural cell attachment and neurite extension in the absence of biochemical modification (Mei et al., 2022). Therefore, PEG systems are commonly functionalized with ECM-derived ligands, including RGD peptides, laminin-derived sequences, or growth factors, to support neural differentiation and network formation (Mei et al., 2022; Swartzlander et al., 2015). Such modifications allow independent control of mechanical and biological signaling within engineered neural microenvironments.

Emerging polymers such as Poly(2-Oxazoline) (POX) further expand the design flexibility of synthetic bioinks. POX-based systems exhibit tunable hydrophilicity, thermoresponsive behavior, and adaptable polymer architectures, enabling the development of dynamically responsive biomaterials (Iminova et al., 2026; Hwang et al., 2013; Mees and Hoogenboom, 2015; Haider et al., 2021; Nemati Mahand et al., 2022; Vanhoeijen et al., 2025). Although their application in neural tissue engineering remains relatively early-stage, POX hydrogels have demonstrated promising cytocompatibility and functional versatility, positioning them as potential next-generation platforms for adaptive and stimuli-responsive neural bioinks (Iminova et al., 2026; Hwang et al., 2013; Mees and Hoogenboom, 2015; Haider et al., 2021; Nemati Mahand et al., 2022; Vanhoeijen et al., 2025; Lusina et al., 2022; Platen et al., 2015; Nemati Mahand et al., 2023). Such tunable biomaterial systems can be employed to design both precise mechanical properties and immunogenic responses in disease models that deal with highly immune response dependent diseases which also present mechanical pathologies in microstructural level such as AD, MG, MS and ALS.

#### 3.3.2 Mechanically supportive polymers

Mechanically supportive synthetic polymers are primarily used to provide structural integrity and long-term stability within neural biofabrication systems. Unlike ultra-soft hydrogel matrices that prioritize cellular permissiveness, materials such as polyurethane (PU) and polycaprolactone (PCL) contribute mechanical resilience, architectural guidance, and fabrication robustness (Ho and hui Hsu, 2018; Janmohammadi and Nourbakhsh, 2019; Yuan et al., 2024; Cheng et al., 2023; Nabipour et al., 2024; Grzesiak et al., 2015; Hsieh et al., 2015).

PU systems are particularly attractive because their segmented polymer structure enables broad tunability of elasticity, toughness, and degradation behavior (Ho and hui Hsu, 2018; Yuan et al., 2024; Cheng et al., 2023). In neural tissue engineering, biodegradable PU formulations have been shown to support neural stem cell viability and neurite extension when combined with surface modifications or bioactive coatings (Nabipour et al., 2024). In addition, conductive and self-healing PU-based materials have emerged as promising candidates for bioelectronic and dynamic neural interfaces (Grzesiak et al., 2015; Hsieh et al., 2015).

PCL, in contrast, is primarily employed as a thermoplastic structural framework fabricated through melt-based extrusion techniques (Janmohammadi and Nourbakhsh, 2019; Liang et al., 2024). Due to its relatively high stiffness, PCL is generally unsuitable as a direct cell-encapsulating matrix for neural cells. Instead, it is commonly used to create mechanically stable support architectures that guide tissue organization and maintain long-term structural integrity. Surface functionalization with ECM proteins such as collagen or laminin is often required to improve cellular interactions and integration with softer hydrogel environments (Weiss et al., 2025; Entekhabi et al., 2016). Consequently, PCL-based systems are most effective when combined with compliant hydrogel matrices that provide the biologically relevant neural microenvironment.

#### 3.3.3 Conductive and electroactive biomaterials

Electrical signaling is a defining functional feature of neural tissue, making conductive biomaterials an increasingly important component of advanced neural biofabrication strategies (Li et al., 2026). Conventional hydrogels are electrically insulating and therefore unable to support efficient electrical communication between developing neural networks. To address this limitation, conductive polymers and nanomaterials are incorporated into bioinks to facilitate electrical signal propagation and enhance neuronal maturation.

Commonly investigated conductive materials include polypyrrole (PPy), polyaniline (PANI), and poly(3,4-ethylenedioxythiophene) (PEDOT), as well as graphene derivatives and carbon-based nanomaterials (Byrne et al., 2026; Cellot et al., 2016; Wang et al., 2026; Bianchi et al., 2023). These components are typically integrated into hydrogel systems as dispersed conductive phases, enabling electrical functionality while preserving the soft mechanical properties required for neural viability. Studies have demonstrated that conductive biomaterials can enhance neurite extension, promote neuronal differentiation, increase synaptic connectivity, and improve electrophysiological activity within engineered neural tissues (Li et al., 2026; Byrne et al., 2026; Cellot et al., 2016; Wang et al., 2026; Bianchi et al., 2023; Aboagye et al., 2026; Firuzeh et al., 2026; Li et al., 2025; Fatimi et al., 2022).

Despite these advantages, significant challenges remain in balancing conductivity, cytocompatibility, and material homogeneity. High concentrations of conductive components may alter hydrogel mechanics, introduce aggregation, or generate cytotoxic effects that negatively influence neural behavior (Byrne et al., 2026; Aboagye et al., 2026). Consequently, conductive systems are most effective when incorporated as multifunctional additives within composite bioinks that integrate electrical responsiveness with controlled mechanical and biochemical microenvironments.

### 3.4 Hybrid and composite bioinks

Hybrid and composite bioinks have emerged as a cornerstone strategy in neural 3D bioprinting, enabling the integration of complementary material properties that cannot be achieved using single-component systems. This integration allows mechanical and biochemical codes of neural microenvironments to be engineered independently. Hybrid systems overcome this limitation by assigning distinct roles to different components, such as using a synthetic polymer network to provide mechanical support while incorporating natural biomaterials to deliver cell-adhesive ligands and biochemical signals (Crosby et al., 2022). This modular design strategy enables more precise control over the cellular microenvironment, which is particularly critical in neural tissue engineering, where cells are highly sensitive to subtle changes in matrix properties.

One of the most widely employed approaches in hybrid bioink design is the formation of interpenetrating polymer networks (IPNs) allowing independent tuning of mechanical properties, degradation behavior, and bioactivity (Fares et al., 2018; Safaei-Yaraziz et al., 2021). Commonly used combinations include GelMA–PEG, hyaluronic acid–alginate, and collagen–synthetic polymer blends, where the synthetic component enhances structural stability and bioprintability, while the natural component supports cell adhesion, differentiation, and neurite outgrowth (Yue et al., 2025).

Hybrid bioinks also enable the incorporation of functional additives. The integration of nanoparticles, growth factors, and bioactive molecules allows fine-tuning of cellular responses, including differentiation, migration, and network formation (Rinoldi et al., 2021). In neural applications, conductive nanomaterials such as graphene derivatives, gold nanoparticles, and conductive polymers have been incorporated to enhance electrical signaling and promote neuronal maturation, thereby improving functional outcomes (Ławkowska et al., 2021; Lee et al., 2015; Moghaddasi et al., 2025).

Another important feature of hybrid systems is their ability to support spatial and compositional heterogeneity. Neural tissues are inherently heterogeneous, consisting of multiple cell types and region-specific extracellular environments. Multi-material bioprinting approaches using hybrid bioinks enable the biofabrication of constructs with spatially defined gradients in stiffness, ligand density, and biochemical composition (Li et al., 2025). This capability is essential for modeling complex neural architectures (Li et al., 2025; Wang et al., 2023).

Hybrid bioinks also play a crucial role in overcoming the bioprintability limitations of ultra-soft, biologically permissive hydrogels. Approaches such as embedded and support-based bioprinting enable deposition of low-viscosity bioinks while maintaining structural fidelity (Yue et al., 2025). These strategies decouple fabrication constraints from biological requirements.

Despite their advantages, hybrid systems introduce additional complexity in material design and processing. Challenges include achieving homogeneous distribution of components, controlling phase separation, and ensuring reproducibility across batches (Crosby et al., 2022; Echalier et al., 2017). These interactions require careful optimization to ensure predictable performance (Echalier et al., 2017).

### 3.5 Dynamic and stimuli-responsive biomaterials

Native neural tissues are highly dynamic systems that undergo continuous structural and biochemical remodeling during development, maturation, and disease progression. Conventional static biomaterials are inherently limited in their ability to capture this temporal complexity, leading to growing interest in dynamic and stimuli-responsive biomaterials that can adapt to changing environmental or externally applied cues (Liao et al., 2025). This shift reflects the need to incorporate time-dependent control into engineered neural systems; which in turn extends the NMDC framework into temporal dimension where mechanical, biochemical and electrophysical codes change over time in response to engineered stimuli.

Stimuli-responsive biomaterials are designed to alter their physical or chemical properties in response to specific triggers, including temperature, pH, enzymatic activity, light, or electrical stimulation (Wang et al., 2025). These materials can exhibit changes in stiffness, porosity, degradation rate, or biochemical ligand presentation, enabling dynamic regulation of the cellular microenvironment (Vats and Benoit, 2013; Gelmi and Schutt, 2021). Such adaptability is essential for regulating time-dependent cellular processes in neural systems (Gelmi and Schutt, 2021).

Thermoresponsive polymers represent one of the most widely studied classes of dynamic biomaterials (Rahimnejad et al., 2022; Klouda, 2015; Zhang et al., 2021). Materials such as poly(N-isopropylacrylamide) (PNIPAM) undergo reversible phase transitions in response to temperature changes, enabling controlled gelation and modulation of material properties (Zhang et al., 2021; Zhang et al., 2004). These systems can facilitate minimally invasive delivery, enable temporal control over matrix stiffness, and support dynamic cell encapsulation strategies (Zhang et al., 2004; Xu et al., 2007; Ekerdt et al., 2018; Han et al., 2016).

Enzymatically responsive biomaterials provide another important mechanism for introducing dynamic behavior. Hydrogels engineered with matrix metalloproteinase (MMP)-sensitive linkers allow cells to locally degrade and remodel the surrounding matrix (Goetsch et al., 2015; Lévesque and Shoichet, 2007). This supports cell-mediated remodeling processes such as neurite extension and migration (Fujioka et al., 2012). Such materials enable a more physiologically relevant interaction between cells and their microenvironment, closely mimicking in vivo conditions (Kharkar et al., 2013). These systems more closely replicate native cell–ECM interactions.

Photoresponsive biomaterials offer precise spatiotemporal control over material properties through light-mediated reactions. By incorporating photolabile crosslinkers or light-sensitive functional groups, it is possible to dynamically modulate stiffness, create microchannels, or release bioactive molecules with high spatial resolution (Zhu et al., 2020). These capabilities enable high-resolution control of neural patterning and microenvironmental cues .

Electro-responsive biomaterials further expand the functional landscape by enabling direct interaction with electrical stimuli, which are inherently relevant to neural tissues. These materials can be used to modulate cellular activity, enhance neuronal differentiation, and promote synchronized network behavior (Kaur et al., 2015). They are particularly relevant for bioelectronic integration and functional neural models (Kaur et al., 2015; Roth et al., 2021).

Despite their significant potential, dynamic biomaterials present several challenges. These include increased complexity in material synthesis, potential cytotoxicity associated with responsive components, and difficulties in achieving precise and reproducible control over dynamic responses (Vats and Benoit, 2013). Integration into bioprinting workflows further requires careful optimization of processing and crosslinking conditions (Liao et al., 2025).

Nevertheless, dynamic and stimuli-responsive biomaterials represent a key strategy toward more physiologically relevant neural tissue models. By enabling temporal and spatial control over the cellular microenvironment, these systems provide powerful tools for studying neural development, disease progression, and regenerative processes (Liao et al., 2025).

### 3.6 3D bioprinting technologies for neural tissue engineering

In neural bioprinting, bioprinting modality and bioink design must be co-optimized to achieve both structural fidelity and biological functionality. Unlike conventional tissue engineering approaches, where materials can often be selected independently of fabrication strategy, neural bioprinting requires integration of bioink rheology, crosslinking mechanisms, and bioprinting technique.

#### 3.6.1 Extrusion-based bioprinting (EBB)

EBB remains the most widely used modality in neural tissue engineering due to its versatility and compatibility with a broad range of bioinks, including high-viscosity, cell-laden hydrogels (Fig. 2A). It enables the fabrication of structurally stable 3D constructs suitable for neural tissue models (Schwab et al., 2020).

**Fig. 2:**
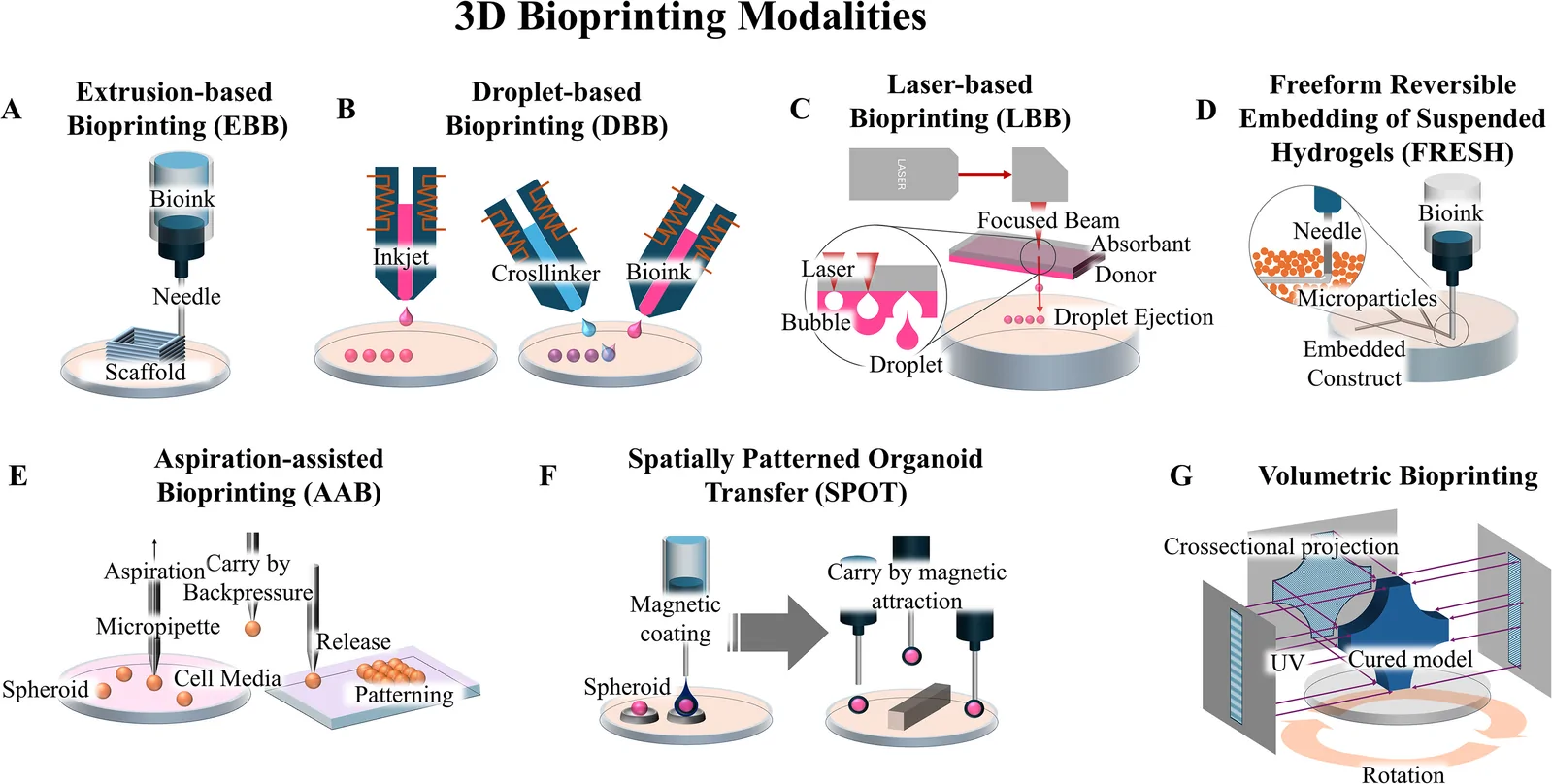
3D Bioprinting Modalities. (A) Extrusion-based bioprinting (EBB), where continuous bioink filaments are deposited through a nozzle to build scaffolded structures. (B) Droplet-based bioprinting (DBB), including inkjet-like systems that dispense discrete droplets of bioink and crosslinker. (C) Laser-based bioprinting (LBB), where a focused laser induces localized droplet ejection from a donor substrate. (D) Freeform Reversible Embedding of Suspended Hydrogels (FRESH), enabling printing within a supportive bath for complex, soft structures. (E) Aspiration-assisted bioprinting (AAB), which uses micropipette aspiration to position cellular spheroids. (F) Spatially patterned organoid transfer (SPOT), utilizing magnetic guidance to arrange organoids in defined positions. (G) Volumetric bioprinting, where 3D constructs are rapidly fabricated with projected light patterns and photopolymerization.

A key advantage of extrusion systems lies in their ability to process shear-thinning materials, which exhibit reduced viscosity under applied stress and rapidly recover their structure after deposition. This allows the use of soft, cell-friendly bioinks while maintaining bioprint fidelity (Ho and hui Hsu, 2018). However, extrusion inherently exposes cells to shear stress during bioprinting, which can negatively impact cell viability and function, especially for neural cells that are highly sensitive to mechanical damage (McCauley et al., 2025). As a result, careful optimization of nozzle geometry, bioprinting pressure, and bioink rheology is required to balance bioprintability and cell survival (Ning et al., 2018).

Recent advances in extrusion-based systems include co-axial bioprinting for generating core–shell structures, gradient bioprinting for spatial modulation of stiffness and composition, and microfluidic-assisted extrusion for improved resolution and multi-material integration (Wang et al., 2025). These strategies enable fabrication of more spatially controlled and physiologically relevant neural constructs.

#### 3.6.2 Droplet-based bioprinting (DBB)

Droplet-based bioprinting (DBB) encompasses a range of techniques that generate discrete droplets of bioink using thermal, piezoelectric, or electrohydrodynamic mechanisms (Fig. 2B). These systems offer high resolution and throughput, typically in the range of 20–100 µm, making them suitable for applications requiring precise spatial patterning, such as microtissue fabrication and organ-on-chip systems. This makes DBB particularly useful for microscale neural patterning.

However, DBB requires low viscosity bioinks, limiting compatibility with many neural hydrogels. This constraint reduces structural stability and limits the fabrication of larger or mechanically robust constructs.

Advanced DBB, such as reactive jet impingement and electrohydrodynamic bioprinting, have been developed to improve control over droplet formation and deposition, enabling the fabrication of heterogeneous constructs with improved precision (Long Ng et al., 2024). Despite these advances, a fundamental trade-off between resolution and material versatility remains.

#### 3.6.3 Laser-based bioprinting (LBB)

Laser-based bioprinting (LBB), particularly laser-induced forward transfer (LIFT), offers a nozzle-free approach for high-resolution cell and material deposition (Fig. 2C). By using laser pulses to transfer bioink droplets from a donor substrate to a receiving surface, LBB enables precise patterning with minimal mechanical stress on cells.

This low-shear environment is particularly advantageous for neural cells, which are highly sensitive to mechanical forces. As a result, LBB systems often achieve high cell viability and enable the precise placement of delicate cell types, including stem cells and primary neurons (Qu et al., 2021). Furthermore, LBB allows fine control over droplet size and deposition patterns, facilitating the fabrication of layered and spatially organized constructs (Shakil Arman et al., 2023).

LBB systems are typically more complex and less accessible than EBB, requiring specialized equipment and careful optimization of laser parameters. Additionally, limited scalability and throughput restrict their use in large-scale tissue fabrication. Moreover, LBB requires low viscosity bioinks, limiting compatibility with many structurally robust hydrogel systems.

#### 3.6.4 Embedded and support bath bioprinting

Embedded bioprinting has emerged as a powerful strategy for fabricating soft and structurally complex neural constructs that are difficult to achieve using conventional freeform bioprinting approaches. In this method, bioinks are deposited within a supportive yield-stress or shear-thinning medium that temporarily stabilizes bioprinted structures during fabrication (Hinton et al., 2015; Lee et al., 2019). This strategy enables decoupling of bioprintability from bioink stiffness, allowing fabrication of structurally stable constructs while preserving the ultra-soft mechanical environment required for neural cell function.

A prominent example is the Freeform Reversible Embedding of Suspended Hydrogels (FRESH) technique, in which bioinks are printed within a gelatin microparticle support bath that can later be removed under mild conditions (Fig. 2D) (Hinton et al., 2015). FRESH enables fabrication of highly compliant constructs with complex geometries, including overhangs, branched structures, and hollow architectures that are otherwise difficult to generate using standard bioprinting methods (Shiwarski et al., 2025). By supporting the use of ultra-soft and low-viscosity bioinks, FRESH-based systems provide improved platforms for reproducing the mechanical properties of native brain tissue.

Recent extensions of embedded bioprinting include the use of spheroid-based support matrices and organoid building blocks, enabling hierarchical tissue assembly, multicellular organization, and perfusable architectures (Shiwarski et al., 2021). Despite these advantages, challenges related to support bath removal, nutrient diffusion, long-term tissue maturation, and process scalability remain important limitations for translational neural applications (Skylar-Scott et al., 2019). Furthermore, shear stress generated during nozzle movement and extrusion remains a significant concern, particularly for fragile neural cell populations, as excessive mechanical forces can reduce cell viability and impair neuronal maturation and network formation.

#### 3.6.5 Emerging bioprinting paradigms for neural systems

Recent advances in bioprinting have introduced a new class of fabrication strategies that move beyond traditional bioink deposition toward cell-centric assembly approaches. These emerging paradigms focus on the spatial organization of pre-formed cellular structures, such as spheroids and organoids, rather than the direct bioprinting of homogeneous hydrogel matrices (Fang et al., 2023). This represents a shift toward architecture-driven tissue assembly.

Aspiration-assisted bioprinting (AAB) represents one such approach, enabling the precise manipulation and placement of individual cell aggregates using controlled aspiration forces (Fig. 2E). AAB minimizes shear stress and allows the assembly of scaffold-free tissues with high spatial accuracy (Ayan et al., 2020). This enables preservation of cell–cell interactions during biofabrication. This technique is promising for neural applications, where preserving cell–cell interactions and maintaining the integrity of delicate multi-cellular structures are critical (Ayan et al., 2020; Ayan et al., 2020; Heo et al., 2020; Ayan et al., 2020; Wu et al., 2020; Dey et al., 2021).

Similarly, recent developments in assembloid bioprinting platforms have demonstrated the ability to spatially organize organoids with high precision, enabling the construction of complex multi-cellular systems that more closely resemble native tissue architecture. These systems allow controlled positioning, fusion, and interaction of distinct cellular modules, facilitating the study of inter-regional neural interactions and disease-specific microenvironments (Roth et al., 2023). This enables modeling of higher-order tissue organization.

Novel 3D bioprinting strategies also improve capabilities in terms of spatial geometry manufacturing of living tissue models. Volumetric bioprinting (VBP) allows for rapid 3D bioprinting of complex geometries using rotary stereolithography (Fig. 2G) (Xie et al., 2026; Bernal et al., 2019). VBP is particularly beneficial in terms of production times for centimeter scale models with micron level resolution.

Collectively, these approaches represent a conceptual shift from bioink-centric fabrication to cell-centric assembly in which the primary building blocks are pre-formed cellular units rather than uniform biomaterial matrices. This is particularly relevant for neural systems, where function depends on higher-order cellular organization (Meng et al., 2025).

While these emerging systems offer significant advantages in terms of biological relevance and precision, they also introduce new challenges related to scalability, automation, and integration with existing bioprinting workflows.

## 4 Engineering the neural microenvironment

The successful engineering of functional neural tissues requires a fundamental shift from conventional scaffold design toward the creation of an active and integrated microenvironment (Gjorevski et al., 2016). In native neural systems, cellular identity, organization, and function are not solely dictated by genetic programs or soluble factors but emerge from continuous interactions with a highly dynamic and multi-physical extracellular milieu (Engler et al., 2006; Jain et al., 2020). Therefore, engineered neural matrices cannot be considered passive structural supports; rather, they must be designed as instructive systems that actively regulate cellular behavior (Discher et al., 2005).

Critically, the neural microenvironment is governed by the interplay of mechanical, electrical, and biochemical cues that operate across multiple spatial and temporal scales (Fig. 3) (Lam et al., 2019). These cues are tightly coupled and co-regulate processes such as neurogenesis, synaptic maturation, and network formation (Liu et al., 2026). Accordingly, next-generation biomaterials must co-engineer mechanical, electrical, biochemical and immune/barrier codes rather than treat them as independent design choices (Cassel De Camps et al., 2021; Tang et al., 2026).

**Fig. 3:**
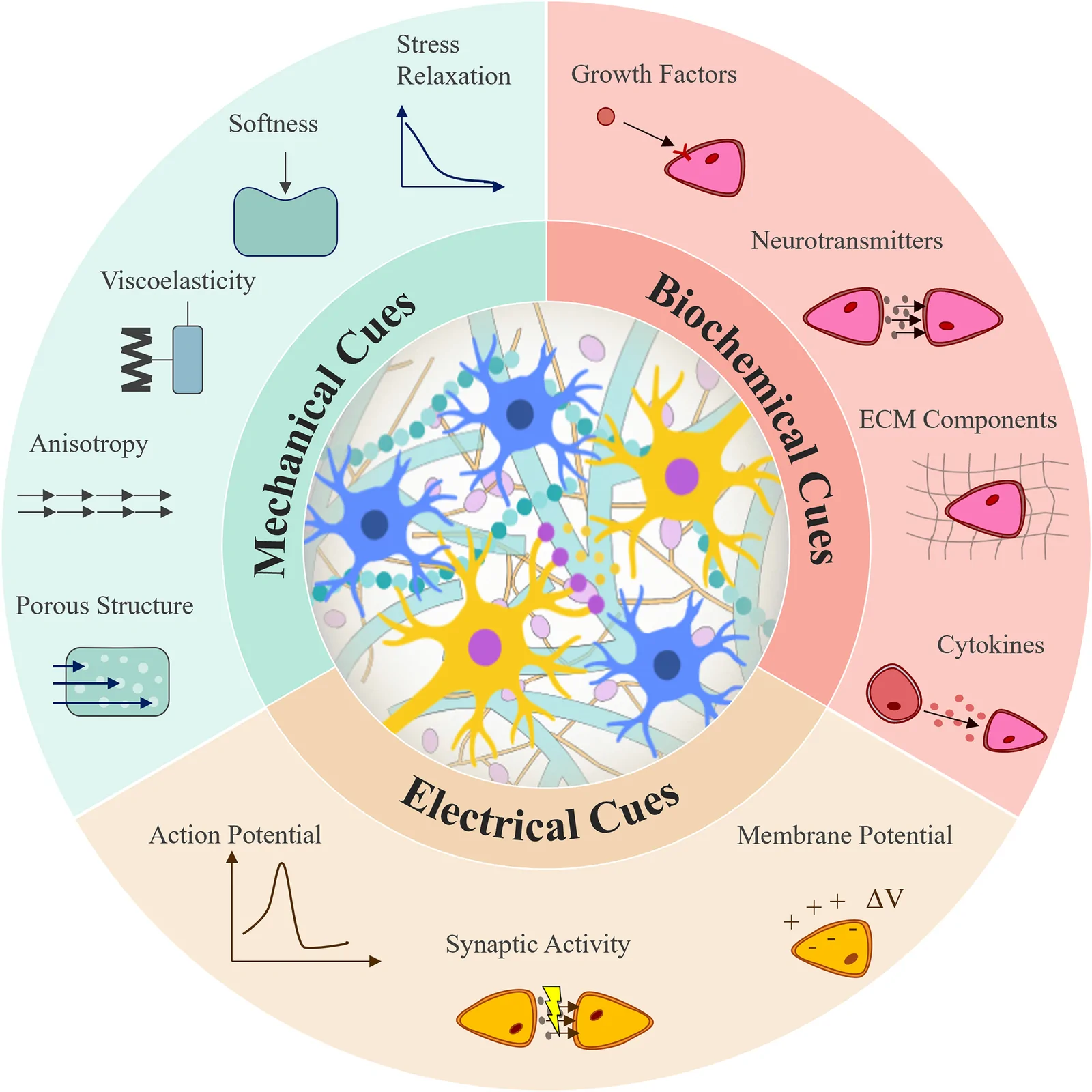
Schematic representation of microenvironmental cues relevant to neural cells. Mechanical cues include stress relaxation, matrix stiffness (softness), viscoelasticity, anisotropy, and porous architecture. Biochemical cues comprise growth factors, neurotransmitters, ECM components, and cytokines. Electrical cues include membrane potential, action potentials, and synaptic activity.

The following sections directly implement the NMDC framework to discuss how mechanical, electrical, immune-responsive, and barrier-mimetic properties can be rationally engineered to reconstruct the neural microenvironment in vitro.

### 4.1 Mechanical microenvironment

This section adresses the mechanical code of NMDC, detailing how matrix stiffness, viscoelacticity, and topography regulate neural and glial cell behaviour/fate. Mechanical cues are among the most dominant regulators of neural cell behavior, acting upstream of biochemical signaling pathways to define cellular identity, morphology, and function (Koser et al., 2016). Neural cells actively sense and respond to their physical environment through mechanotransuction pathways such as YAP/TAZ signaling (Dupont et al., 2011). Consequently, the mechanical properties of engineered scaffolds are not merely structural parameters, but primary determinants of neural tissue formation (Song and Dityatev, 2018).

A defining characteristic of the central nervous system is its extreme softness, with an elastic modulus typically ranging from 0.1 to 2 kPa depending on anatomical region and cell composition (Budday et al., 2019). Neural stem and progenitor cells encapsulated within compliant hydrogels preferentially undergo neurogenesis, exhibiting enhanced neurite extension and synaptic connectivity (Mattiassi et al., 2023). Thus, the precise tuning of matrix stiffness represents a foundational design parameter for engineering neuron-dominant tissues.

Beyond stiffness, the viscoelastic nature of the neural ECM plays a critical role in regulating cell behavior (Franze et al., 2013). Conventional covalently crosslinked hydrogels often create restrictive environments that limit cell spreading and neurite outgrowth (Roth et al., 2023; Huang et al., 2025). In contrast, viscoelastic hydrogels permit cells to remodel their surroundings even in non-degradable matrices (Sanazzaro et al., 2026; Tang et al., 2021). This highlights the importance of stress relaxation and dynamic remodeling in neural biomaterial design.

Mechanical properties also critically influence neuroimmune behavior, which is a property relating mechanical code to immune/barrier code. Microglia, the resident immune cells of the CNS, are highly sensitive to matrix stiffness and viscoelasticity (Ayata and Schaefer, 2020). In stiff or non-relaxing environments, microglia adopt an activated, pro-inflammatory phenotype (Liu et al., 2023). Conversely, soft matrices support the maintenance of a ramified, homeostatic phenotype, enabling continuous surveillance of the surrounding microenvironment (Blaschke et al., 2020). These observations highlight the necessity of mechanically permissive environments not only for neuronal function but also for establishing physiologically relevant neuroimmune interactions.

In addition to bulk mechanical properties, microscale and nanoscale topographical features provide further layers of control over neural organization (Severs et al., 2024). Anisotropic structures, such as aligned nanofibers or nano-gratings, guide neurite extension and promote neuronal differentiation through integrin-mediated focal adhesion signaling (Lim et al., 2010). In contrast, discontinuous topographies, including micropillar arrays, can modulate astrocyte differentiation or maintain microglial homeostasis (Sharaf et al., 2022; Ankam et al., 2013). The dimensional scale of these features further influences cellular responses, with structures mimicking radial glia or microvascular dimensions eliciting distinct morphogenetic outcomes (Czeisler et al., 2016).

Finally, mechanical cues operate in concert with biochemical signals embedded within the matrix, again bridging between two central codes of NMDC framework. HA-based hydrogels, for instance, interact with receptors such as CD44 and RHAMM (Receptor for Hyaluronan Mediated Motility) to support neural progenitor function, while synthetic peptide motifs derived from ECM proteins, including IKVAV and YIGSR, enhance neurite outgrowth and neuronal survival (Ma et al., 2023; Wilson et al., 2020; Jain and Roy, 2020). Together, these mechanical and biochemical design parameters establish a highly instructive microenvironment that governs neural tissue development at multiple levels.

### 4.2 Conductive and active environments

This section adresses the electro-physical code of the NMDC framework, focusing on how conductivity and mechanical forces (eg. shear stress) complementarily regulate neural network activity. A fundamental limitation of conventional hydrogels is their intrinsic electrical insulation, which restricts the propagation of bioelectric signals essential for neural function. Neurons communicate through tightly regulated ionic fluxes and action potentials; therefore, a permissive electrical microenvironment is critical for the emergence of synchronized network activity and functional synaptic connectivity (Tang-Schomer et al., 2014).

To overcome this limitation, conductive hydrogels have been developed by incorporating electrically active nanomaterials such as graphene, carbon nanotubes (CNTs), and black phosphorus (Gupta and Lahiri, 2016; Chen et al., 2025). Importantly, neural tissues rely primarily on ionic conduction, whereas most engineered conductive materials operate through electronic charge transport (Balint et al., 2014).

Within this framework, conductive biomaterials act not merely as passive signal conduits but as facilitators of functional network maturation. For example, collagen-based hydrogels integrated with CNTs, or conductive polymers have been shown to enhance neural stem cell proliferation and differentiation, accelerate neurite outgrowth, and promote the formation of interconnected neural networks (Gupta and Lahiri, 2016; Malarkey et al., 2009; Ranjbar et al., 2023). Similarly, graphene-containing hydrogels support increased synaptic density, higher firing frequencies, and improved network synchronization compared to electrically insulating matrices (Martín et al., 2017; Guo et al., 2016). However, the ultimate benchmark of these systems is not conductivity alone, but their ability to support coordinated electrophysiological activity, as demonstrated through techniques such as patch-clamp recording, calcium imaging, and multi-electrode array (MEA) analysis (Frega et al., 2014; Maki et al., 2014).

Beyond passive conductivity, emerging classes of electrically active biomaterials enable dynamic signal generation in response to external or cell-driven stimuli. Piezoelectric polymers and nanostructures, for instance, convert mechanical deformation into localized electrical potentials, providing a mechanism for self-stimulating neural constructs (Shen et al., 2025). This mechano-electrical coupling has been shown to enhance neurite extension and promote (Thampi et al., 2020; Wang et al., 2025).

In parallel, the neural microenvironment is continuously shaped by dynamic physical forces, including fluid flow and shear stress, particularly within the context of cerebrospinal fluid circulation and the BBB (Castillo Ransanz et al., 2022; Park et al., 2017). Controlled fluid shear stress has been shown to enhance endothelial barrier integrity, regulate astrocyte morphology, and modulate microglial activation states, thereby influencing neuroimmune interactions (Wakida et al., 2020).

Collectively, conductive and active biomaterials represent a critical step toward recapitulating the electromechanical complexity of the native neural microenvironment. These findings highlight the need to integrate electrical, mechanical and fluid cues within engineered systems, moving beyond static scaffolds toward dynamic and functionally responsive neural microenvironment.

### 4.3 Immune responsive materials

This section addresses the immune code of the NMDC framework by describing biomaterial strategies that sense and regulate microglial and astrocytic activation.The neural microenvironment is not immunologically inert but is instead governed by a highly dynamic and tightly regulated neuroimmune network. Resident glial populations, including microglia and astrocytes, interact continuously with neurons, infiltrating immune cells, and soluble signaling molecules to maintain homeostasis and respond to injury (Wang et al., 2025; Yang et al., 2025).

Microglia, the primary immune effector cells of the central nervous system, exhibit remarkable phenotypic plasticity, transitioning between homeostatic surveillance states and activated, pro-inflammatory phenotypes in response to environmental cues (Wang et al., 2025). Astrocytes similarly display context-dependent behavior, supporting neuronal metabolism and synaptic regulation under physiological conditions, while adopting neurotoxic phenotypes under inflammatory stress (Liddelow et al., 2017). As such, biomaterials designed for neural applications must not only support cellular viability but actively interface with and modulate these neuroimmune processes.

Immune-responsive biomaterials have emerged as a class of adaptive systems capable of sensing pathological signals and responding in a controlled and programmable manner (Tian et al., 2026). Reactive oxygen species (ROS) responsive hydrogels represent one of the most extensively studied platforms in this domain (Tian et al., 2026; Ren et al., 2022). In the context of neural tissue engineering, such responsiveness is particularly critical, as neuroinflammation is not a secondary effect but a primary driver of tissue dysfunction and degeneration.

By incorporating ROS-sensitive chemical moieties, biomaterials can undergo selective degradation or structural transformation in oxidative environments, enabling on-demand drug release and simultaneous ROS scavenging (Li et al., 2023; Chen et al., 2025; Cao et al., 2026).

Cytokine-responsive systems offer an additional layer of specificity by targeting key inflammatory signaling pathways. Materials designed to respond to elevated levels of cytokines such as IL-1β and TNF-α can regulate local immune activity either through cytokine-triggered degradation or via the controlled release of anti-inflammatory agents and biologics. In neural systems, such approaches hold promise for interrupting self-sustaining inflammatory feedback loops that exacerbate neuronal damage (Gao et al., 2023).

Enzyme-responsive biomaterials provide perhaps the highest degree of biological specificity, leveraging the selective activity of enzymes that are upregulated in pathological states. In neural tissue engineering, MMP-sensitive hydrogels have been shown to facilitate gradual matrix turnover, allowing for dynamic tissue integration while synchronizing scaffold degradation with cellular activity.

Importantly, immune-responsive materials should not be viewed solely as drug delivery platforms, but as regulatory interfaces within a complex neuroimmune feedback system. The ability to sense, interpret, and respond to local immune signals positions these biomaterials as active participants in shaping tissue-level outcomes. By modulating the balance between inflammatory and regenerative processes, these systems can influence neuronal survival, synaptic stability, and long-term functional integration (Galindo et al., 2024).

Looking forward, the integration of immune-responsive behavior with mechanical, electrical, and biochemical design parameters will be essential for constructing truly biomimetic neural environments (Peressotti et al., 2021). Such convergence will enable the development of adaptive, self-regulating biomaterial systems capable of maintaining homeostasis, responding to pathological perturbations, and ultimately advancing both in vitro disease modeling and translational therapeutic strategies for neurodegenerative disorders.

### 4.4 Barrier mimetic materials

This section addresses the barrier component of the NMDC immune/barrier code, focusing on biomaterial strategies that mimic BBB structure and selective transport. BBB represents one of the most stringent physiological interfaces in the human body, maintaining CNS homeostasis by tightly regulating molecular and cellular transport between the bloodstream and neural tissue (Konig et al., 2025). While this selective permeability is essential for normal brain function, it also constitutes a major bottleneck in therapeutic development, as more than 98% of small-molecule drugs and nearly all biologics fail to effectively cross the BBB. Consequently, the development of biomimetic platforms that accurately recapitulate BBB structure and transport dynamics has become a central challenge in neural tissue engineering and drug discovery (Potente and Mäkinen, 2017).

Structurally, the BBB is formed by a continuous monolayer of brain microvascular endothelial cells (BMECs) interconnected by tight junction proteins such as claudin-5 and occludin and supported by pericytes and astrocytes that together comprise the neurovascular unit (Greene et al., 2019). These cells are embedded within a specialized basement membrane rich in ECM proteins, including laminin, collagen IV, and fibronectin, which collectively regulate barrier integrity, mechanotransduction, and cellular signaling. Disruption of this architecture, as observed in inflammation and neurodegenerative diseases, leads to increased permeability, impaired transport regulation, and subsequent neural dysfunction (Konig et al., 2025).

Traditional 2D BBB models fail to reproduce the complex interplay of mechanical, biochemical, and transport-related cues that define in vivo barrier function. In response, biomimetic hydrogels and 3D scaffolds have emerged as powerful platforms for reconstructing the BBB microenvironment. Natural polymers such as collagen, HA, and fibrin provide biologically active matrices that support endothelial adhesion, proliferation, and tight junction formation (Grifno et al., 2019). When tuned to brain-like stiffness, these materials enable the formation of vascular structures exhibiting physiologically relevant permeability and mechanosensitivity, features not achievable in conventional 2D systems (Guo et al., 2025).

Synthetic hydrogels, particularly those based on PEG, offer enhanced control over matrix properties and biochemical functionalization (Ahmad et al., 2024). By incorporating peptide motifs such as IKVAV and RGD, and integrating MMP-sensitive crosslinking, these systems can mimic key aspects of the basement membrane while enabling dynamic remodeling (Ahmad et al., 2024). Co-culture of ECs with astrocytes and pericytes within such engineered matrices has been shown to yield robust barrier formation, characterized by increased transendothelial electrical resistance (TEER), reduced permeability to tracer molecules, and upregulation of tight junction proteins (Ahmad et al., 2024; Appelt-Menzel et al., 2017). These findings highlight the importance of coupling structural design with functional validation in BBB models.

Beyond static systems, dynamic and perfusable models have further advanced the physiological relevance of BBB platforms. Microfluidic and organ-on-a-chip systems enable precise control over fluid shear stress, which is a critical regulator of endothelial phenotype, tight junction maturation, and barrier integrity (Ta et al., 2025). For example, shape-memory biomaterial-based systems that transition from planar to tubular architectures under physiological conditions allow the formation of perfusable vascular networks with polarized transport characteristics (Ta et al., 2025). These platforms enable quantitative analysis of drug permeability, immune cell transmigration, and neurovascular interactions under flow conditions.

Importantly, BBB function is governed by a delicate balance between transport selectivity and permeability. Effective models must therefore not only establish tight barrier properties but also preserve controlled molecular transport, including active efflux mechanisms mediated by transporters such as P-glycoprotein (P-gp). This dual requirement underscores the need for integrated design strategies that combine mechanical tuning, biochemical signaling, and dynamic flow conditions.

Collectively, barrier-mimetic biomaterials have enabled a transition from simplified monolayer systems toward complex, multi-cellular, and dynamic models that more accurately reflect human physiology. By integrating hydrogel-based matrices, peptide functionalization, and microfluidic control, these platforms provide unprecedented opportunities to study BBB function, disease mechanisms, and drug transport (Guo et al., 2025). Future advances will depend on the convergence of barrier engineering with mechanical, electrical, and immune-responsive systems, ultimately enabling the development of fully integrated neural tissue models with high translational relevance (Guo et al., 2025; da Silva et al., 2025).

## 5 Disease modeling applications

The folowing disease-specific sections demonstrate how the main codes within NMDC framework combine differently for neurodegenerative conditions; PD and ALS are driven mainly by biochemical and immune/barrier code disruptions, MG and ALS-associated neuromuscular junction pathology are mostly related to electro-physical code; AD reflects parallel failure of the mechanical, biochemical and immune/barrier codes; and MS is fundamentally an immune/barrier and biochemical code disorder (at lesion level). Matching each disease onto the NMDC framework highlights where current models are successful in mimicking disease phenotype, and where design gaps remain.

### 5.1 PD models

PD pathology is mainly driven by disruption of the biochemical code via alpha-synuclein aggregation and metabolic stress, and immune/barrier code via T-cell mediated neuroinflammation. 3D bioprinted in vitro models have emerged as transformative platforms for studying PD, addressing critical limitations of traditional 2D cultures by enabling the reconstruction of complex cellular interactions and microenvironmental cues. 3D systems allow for the modeling of dopaminergic neuron degeneration within a spatially organized and physiologically relevant context, capturing key pathological hallmarks of PD, including α-synuclein aggregation, neuroinflammation, metabolic dysfunction, and progressive neuronal loss (Zuccoli et al., 2025; Mao et al., 2024; Chiaradia and Lancaster, 2020).

Among these platforms, midbrain organoids derived from induced pluripotent stem cells (iPSCs) have become central to PD research due to their ability to recapitulate early human neurodevelopment and generate functional dopaminergic neurons (Fig. 4A) (Yayon et al., 1991). These organoids consistently express canonical dopaminergic markers and reveal disease-specific transcriptional and metabolic alterations when derived from PD patients, supporting their utility for modeling intrinsic neuronal vulnerability (Velasco et al., 2019) Importantly, recent studies have demonstrated improved reproducibility across independent organoid batches, addressing a long-standing challenge in the field and enhancing their reliability for disease modeling and drug discovery applications (Zuccoli et al., 2025).

**Fig. 4:**
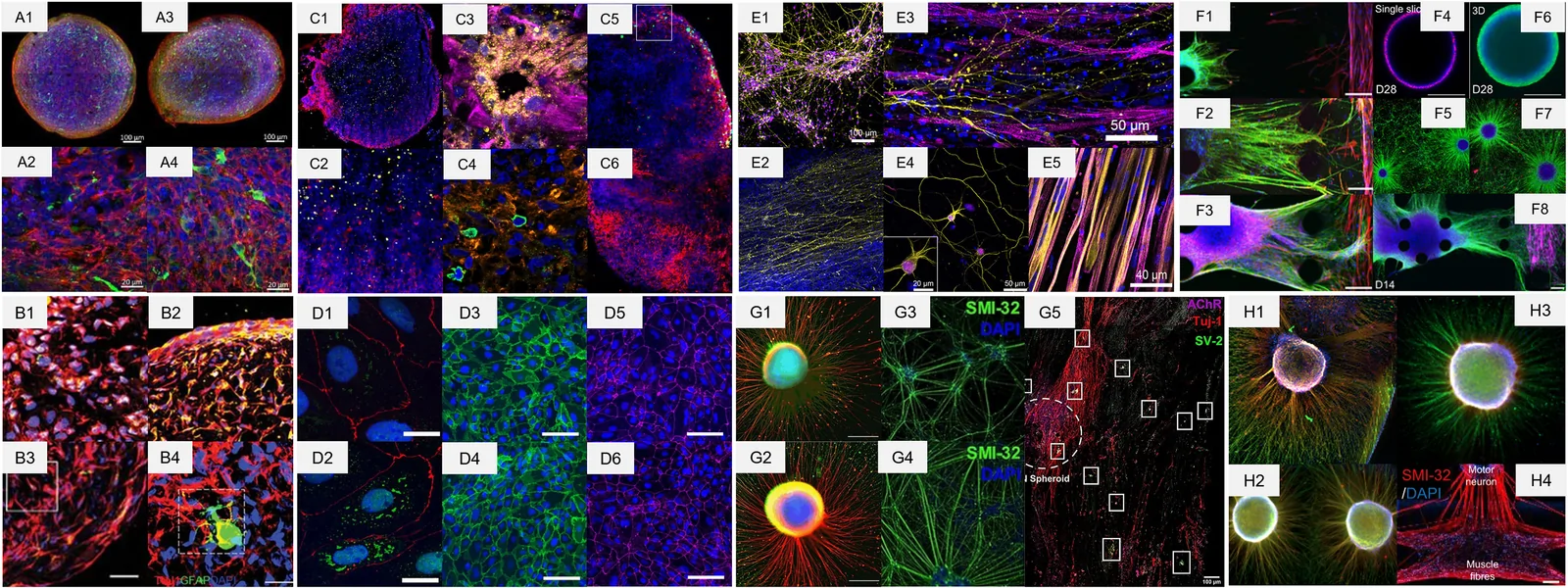
PD Models (A) Mid-brain organoid PD disease model (A1,A3) Confocal images of TH (green), MAP2 (red), and nuclei (blue) of midbrain organoid section staining at day 30 (A2&4) Close section confocal images of TH (green), MAP2 (red), and nuclei (blue) of midbrain organoid section staining at day 30 (B) 3D Induced dopaminergic neuron assembloids (iDA) (B1) Immunofluorescence staining of TH- and VMAT2-positive cells in 3D iDA organoids at 20 days (B2) Immunofluorescence microscopy of TUJ1- and pS129 α-synuclein-positive cells in the presence and absence of α-synuclein (A53T) induction for 20 days (B3) Generation of 3D-iDA neuron-astrocyte assembloids, Immunofluorescence staining of TUJ1- and TH-positive cells within 3D iDA and 3D assembloids treated with DMSO or 0.5 mM MPTP during 3D direct reprogramming (B4) TUJ1- and GFAP-positive cells within control 3D assembloids after 3D direct reprogramming (C) T-Cell integrated mid-brain organoiid PD model (C1-2) Human mid-brain organoid (hMO) at day 7 stained for MAP2 (purple), TUNEL (yellow) and DAPI (blue) (C3-4) Immunostaining of integrins and chemokine ligands in hMO tissue: ICAM-1 (orange), VCAM-1 (yellow), and CXCL12 (red) (C5-6) Observation of decreased MAP2 signal in hMO co-cultured with T cells (C5) in comparison with hMO cultured without T cells (C6) ALS Disease Models (D) Patient derived ALS BBB model for focused ultrasound-mediated anti-TDP-43 antibody delivery (D1-2) Immunofluorescence images of TDP-43 (green) and ZO-1 (red) with Hoechst nuclear counterstaining (blue) of C9orf72 ALS (D1) and sporadic ALS iBECs (D2), scale bar = 20 μm (D3) Immunofluorescence images of Glut-1 (green) with Hoechst nuclear counterstaining (blue) in healthy donor, C9orf72 ALS iBECs (D4) Immunofluorescence images of Glut-1 (green) with Hoechst nuclear counterstaining (blue) in healthy donor, C9orf72 ALS iBECs (D5-6) immunofluorescence images of claudin-5 (magenta) with Hoechst nuclear counterstaining (blue) C9orf72 ALS (D5) and sporadic ALS iBECs (D6), scale bar = 100 μm (E) 3D bioengineered human neuromuscular junctions to model ALS (E1) iPSC-derived Neurons stained for Islet-1 and Tuj1 after 14 days (E2) Neuron muscle co-cultures stained for NF-H and DAPI after 21 days (E3) Immunostained images of a 21 days-old neuromuscular co-culture against Tuj1, α-Actinin and DAPI (E4) Individual iPSC-derived neurons stained for Neurofilament Heavy Chain (NF-H) and Islet-1 after 14 days (E5) NMJ model immunocytochemical stainings of 21 days-old muscle fiber tissue against α-Actinin (purple), Neurofilament Heavy Chain (NF-H) (yellow) and DAPI (blue) (F) Microphysiological 3D model of ALS from human iPSC-derived muscle cells and optogenetic motor neurons (F1-3) Engineered NMJ model (F4-6) Formation of an NSC spheroid and differentiation characterized by the immunostaining of HB9 and 3D reconstruction stained by Tuj1. Scale bars, 200 μm (F5-7) After the formation of NSC spheroids on D20 (F5) and D40 (F7), (F8) NMJ unit on D14 with continuous treatment of glutamic acid. MG Disease Models (G) Bioengineered human motor unit with physiologically functional NMJ model (G1-2) Motor neuron progenitors cultured as 3D spheroids day 7 (G1) and day 21 (G2), (G3-4) Motor neuron progenitor cells cultured in monolayer for 2, 7, 14 and 21 days positive for SMI-32 (G5) Confirmed innervation by confocal tile scan of neuromuscular tissue detailing AChR and SV-2 evidencing multiple synaptic contacts (white boxes) via co-localization of pre- and post-synaptic markers and Tuj-1 + neuronal axons NSC-MN spheroid culture (green, Tuj1; red, islet1). (H)NMJ model to study pathogenic mechanisms of anti-AChR and autoantibody-positive myasthenia gravis (H1-3) Neuron spheroids characterized by the immunostaining of Tuj1, HB9, SMI-32, and VGLUT1. (H4) SMI-32 staining of neuromuscular junction (NMJ) showing innervation of sarcomere structures. The pattern of a sarcomere structure is stained by sarcomeric α-actinin and titin. Scale bar = 200 µm. Panel A adapted from (Zuccoli et al., 2025), B from (Kim et al., 2025), C from (Gerasimova et al., 2025), D from (Wasielewska et al., 2024), E from (Massih et al., 2023), F from (Osaki et al., 2018), G from (Rimington et al., 2021), H from (Shin et al., 2025). All licensed under CC BY.

Beyond genetic forms of PD, 3D organoid systems have also enabled the investigation of idiopathic disease mechanisms. Patient-derived organoids have been shown to reproduce key features of midbrain architecture and dopaminergic network formation, while revealing alterations in early and late neuronal differentiation markers such as LMX1A and tyrosine hydroxyl (Chlebanowska et al., 2020). These findings highlight the capacity of 3D systems to capture subtle developmental and phenotypic differences that are not accessible in conventional models.

Recent advances have further expanded the versatility of 3D PD models through alternative cellular engineering approaches. Direct reprogramming strategies, for instance, enable the rapid generation of induced dopaminergic neurons from patient-derived fibroblasts within organoid or assembloid systems, providing a scalable and patient-specific platform for disease modeling (Kim et al., 2025). Such approaches reduce the time and variability associated with iPSC differentiation while preserving disease-relevant phenotypes.

A critical emerging dimension in PD modeling is the incorporation of immune components. Neuroinflammation is increasingly recognized as a central driver of PD pathology rather than a secondary consequence. Co-culture systems integrating T cells or microglia with midbrain organoids have demonstrated active immune cell infiltration and subsequent dopaminergic neuron loss, providing direct evidence of adaptive and innate immune contributions to disease progression (Fig. 4B) (Gerasimova et al., 2025). These findings underscore the importance of modeling neuroimmune interactions within 3D systems to capture the full complexity of PD pathophysiology.

Complementary to organoids, neurosphere-based models offer a more scalable and experimentally tractable alternative. Derived from neural progenitor cells, these systems enable controlled studies of dopaminergic differentiation and quantitative assessment of α-synuclein accumulation and metabolic alterations (Fig. 4C) (Joshi et al., 2025). Similarly, neurospheres generated from PD patient-derived iPSCs carrying α-synuclein mutations exhibit elevated pathogenic protein levels and altered lipid metabolism, further supporting their relevance for disease modeling (Raja et al., 2022).

Bioprinting technologies have further advanced the field by enabling precise spatial organization of multiple cell types within defined architectures. Recent studies have demonstrated the development of 3D bioprinted PD models incorporating dopaminergic neurons and microglia within tunable hydrogel matrices, such as PEG–HA systems (Mao et al., 2024). Under neurotoxic conditions, these constructs recapitulate key disease features, including mitochondrial dysfunction, increased α-synuclein expression, and robust neuroinflammatory responses. The ability to control cellular distribution and microenvironmental parameters represents a significant advantage over self-organizing systems, enabling more reproducible and customizable disease models. PD disease models benefit greatly from design considerations provided by NMDC framework and biochemical and immune codes. These design considerations emphasize the expression and neuroinflammation aspects in PD models.

Collectively, 3D stem cell-derived models have fundamentally reshaped the study of PD progression by enabling the reconstruction of key pathological features within a controlled in vitro environment. The integration of neuronal, glial, and immune components, combined with advances in reproducibility and bioengineering, positions these systems as powerful platforms for mechanistic studies and therapeutic development.

### 5.2 Myasthenia gravis (MG) and neuromuscular junction disease models

MG is an autoimmune disorder defined by antibody mediated disruption of neuromuscular junction signaling, most commonly through targeting of the acetylcholine receptor, resulting in impaired synaptic transmission and progressive muscle weakness (Lazaridis and Tzartos, 2020; Koneczny and Herbst, 2019). Unlike central neurodegenerative diseases, MG pathology is fundamentally rooted in the structural and functional integrity of the neuromuscular synapse, where precise alignment between motor neurons and muscle fibers governs signal transmission efficiency. Consequently, disease manifestation is highly sensitive to microenvironmental organization, receptor distribution, and synaptic architecture (Kim et al., 2023).

Conventional in vivo and 2D in vitro models have provided valuable insights into immune mediated mechanisms, including complement activation and receptor internalization, yet they fail to recapitulate the spatially organized and mechanically regulated nature of the human neuromuscular junction. Given that synaptic stability and receptor clustering are tightly dependent on cellular alignment and extracellular matrix context, these limitations constrain the ability of traditional systems to accurately model disease progression and therapeutic response.

Recent advances in 3D bioengineered systems have begun to address these challenges by enabling controlled reconstruction of neuromuscular microenvironments. In particular, the ability to spatially organize motor neurons, skeletal muscle fibers, and supporting cell types within defined architectures has revealed how synaptic geometry influences both signal transmission and susceptibility to antibody mediated disruption. These findings emphasize that MG pathology cannot be fully understood without considering the engineered microenvironment in which neuromuscular interactions occur.

Microfluidic based co-culture platforms further extend this capability by introducing spatial compartmentalization and dynamic control over the local microenvironment. In these systems, motor neuron spheroids and human skeletal muscle fibers are cultured within ECM mimicking hydrogels, enabling the study of antibody mediated complement activation and calcium signaling deficits within a physiologically relevant context (Shin et al., 2025). The incorporation of controlled fluidic environments allows modulation of signaling gradients and immune components, offering a more refined representation of disease relevant interactions.

The integration of 3D bioprinting introduces a critical level of architectural precision, enabling deterministic assembly of neuromuscular tissues with defined spatial organization. Bioprinted constructs allow alignment of muscle fibers, controlled placement of motor neurons, and tuning of ECM properties, all of which directly influence synaptic formation and stability. This level of control is particularly relevant in MG, where receptor clustering, synaptic density, and local microenvironmental cues govern antibody binding and downstream functional impairment. In context of NDMC framework, MG models prominently make use of electrophysical and biochemical design codes, mimicking synapse disfunction at NMJ which encompasses the guiding design considerations.

Engineered motor unit models incorporating 3D matrices and bioprinted architectures consistently demonstrate improved neuromuscular junction maturation, including enhanced alignment, receptor clustering, and electrophysiological functionality (Fig.s 4 H-G). Although not exclusively developed for MG, these systems provide powerful platforms for interrogating acetylcholine receptor blockade, complement mediated damage, and synaptic transmission deficits under controlled conditions (Shin et al., 2025; Kim et al., 2020; Vila et al., 2021). Importantly, they enable systematic investigation of how microenvironmental parameters modulate disease severity and progression.

Collectively, these advances position 3D neuromuscular models as essential tools for bridging the gap between simplified in vitro systems and complex human physiology. By integrating biomaterials, stem cell technologies, and biofabrication strategies, these platforms enable mechanistic dissection of antibody mediated synaptic dysfunction while supporting scalable approaches for therapeutic screening (Shin et al., 2025; Kim et al., 2020). Moreover, the ability to engineer patient specific neuromuscular microenvironments offers a promising path toward personalized modelling of MG and the development of targeted treatment strategies.

### 5.3 ALS disease models

In vitro disease models have played a foundational role in elucidating the cellular and molecular mechanisms underlying ALS. While lacking the spatial complexity of 3D systems, 2D cultures, particularly those derived from iPSCs, offer high reproducibility and accessibility for mechanistic investigations. These systems have been instrumental in uncovering non cell autonomous neurodegeneration, glial dysfunction, RNA pathology, and immune contributions to disease progression (Karagiannis and Inoue, 2020; Rothstein et al., 2025).

A central theme emerging from ALS models is the critical role of glial cells in driving motor neuron degeneration. Co-culture systems using iPSC-derived astrocytes and motor neurons demonstrate that astrocytes can induce neuronal death through the secretion of toxic factors and disruption of metabolic support. These findings reinforce the concept that ALS is not solely a neuron specific disease, but rather a multi-cellular pathology shaped by its surrounding microenvironment (Baofengfeng et al., 2022).

Expanding on this, work by Zelic et al. has shown that tri-culture systems incorporating astrocytes, microglia, oligodendrocytes, and motor neurons exhibit pronounced inflammatory signaling mediated through receptor interacting protein kinase 1 pathways, with upregulation of cytokines such as SPP1, CHI3L2, and CCL2. Importantly, inhibition of RIPK1 reshapes inflammatory glial states and delays disease progression, underscoring the therapeutic relevance of targeting multi-cellular interactions (Zelic et al., 2024).

Microglial contributions to ALS pathology have also been extensively characterized using transcriptomic approaches compatible with simplified culture systems. Single nucleus RNA sequencing studies in mouse and primate systems have identified a disease associated microglial subtype that promotes motor neuron vulnerability through inflammatory and lysosomal pathways (Sun et al., 2023). Notably, these microglial states overlap with aging related transcriptional programs, suggesting a shared micro-environmental axis between aging and ALS. Although derived from in vivo systems, these findings inform the design and interpretation of reductionist co culture models, where microglia induced oxidative stress and senescence can be recapitulated and studied in isolation.

Another key insight from ALS models is the importance of RNA binding protein dysfunction, particularly involving TDP 43. Loss of function and aggregation of TDP 43 in cultured cells disrupt RNA splicing and processing across multiple central nervous system cell types (Ling et al., 2015). These studies demonstrate that TDP 43 pathology extends beyond neurons to affect glial and oligodendrocyte function, reinforcing the concept of system wide dysregulation (Fig. 4D). Similarly, iPSC-derived neuronal cultures modeling C9ORF72 (chromosome 9 open reading frame 72) repeat expansions reveal how toxic RNA species and dipeptide repeat proteins drive cellular stress. These models show that abnormal protein accumulation induces heterochromatin remodeling, nuclear lamina disruption, and reduced HP1α levels, linking genetic mutations to epigenetic and structural nuclear defects (Zhang et al., 2019). In context of NDMC framework, such pathway dependent disease properties constitute biochemical and electrophysiological design codes and pertaining considerations, as in neural disease model design, gene upregulation, related receptor activity and signaling needs to be thoroughly considered.

Immune system involvement represents another dimension effectively explored in simplified in vitro systems. Studies have demonstrated that T cells and monocytes can infiltrate the central nervous system and reshape the inflammatory microenvironment, thereby influencing disease progression (Zondler et al., 2016). In parallel, vascular dysfunction has been shown to precede motor neuron loss, facilitating immune cell entry and further exacerbating inflammation (Wasielewska et al., 2024). These findings highlight the importance of incorporating vascular and immune components into engineered models to better capture disease relevant interactions. Thus immune and barrier codes ties into the NMDC framework establishing multi-faceted design considerations encompassing altogether mechanical, biochemical, electro-physical and immune compartment codes.

Beyond the central nervous system, in vitro studies have contributed to an expanded view of ALS as a systemic disease. Recent studies emphasize the active role of skeletal muscle in ALS progression, suggesting that muscle pathology may contribute to motor neuron degeneration rather than merely reflecting it (Fig. 4E). Such perspectives support the development of multi lineage co-culture systems that integrate muscle cells alongside neurons and glial populations (Shefner et al., 2023).

Optogenetic 3D NMJ models exemplify this transition toward functional and controllable systems. By integrating optogenetically responsive iPSC-derived motor neurons with engineered muscle tissues, these platforms enable precise stimulation and real time measurement of contractile responses (Fig. 4F). Exposure to MG patient derived antibodies in such systems reproduces disease specific synaptic dysfunction, providing direct functional readouts of impaired neuromuscular transmission under controlled conditions (Vila et al., 2021).

Despite their strengths, conventional in vitro models remain fundamentally limited by their inability to recapitulate the spatial organization, mechanical context, and transport dynamics of native neural tissues. Disease progression in ALS is not governed solely by cell intrinsic factors but is tightly regulated by the surrounding microenvironment, including ECM properties, cellular architecture, and biochemical gradients. This limitation has driven the emergence of 3D systems designed to better mimic tissue level organization and function (Kasper et al., 2023; Maskell et al., 2026).

Recent advances in 3D ALS models have been particularly transformative in revealing disease mechanisms at the neuromuscular junction. Co-culture systems integrating motor neurons and skeletal muscle have demonstrated that patient derived cells exhibit impaired synaptic transmission and reduced muscle contractility, thereby capturing early functional deficits associated with disease onset (Massih et al., 2023). The use of fibrin hydrogels and polydimethylsiloxane (PDMS)-based architectures has enabled the formation of aligned, contractile muscle fibers innervated by motor neurons, providing a controllable microenvironment for disease modeling and therapeutic testing (Santoso and McCain, 2020; Santoso et al., 2025).

Microfluidic organ-on-chip platforms further extend this capability by introducing spatial compartmentalization and dynamic signaling. In a landmark study, Osaki et al. developed a 3D ALS-on-chip system integrating patient derived motor neuron spheroids and engineered muscle tissues within a PDMS based microfluidic device (Osaki et al., 2018). This model successfully recapitulated key ALS features such as excitotoxicity induced degeneration and reduced contraction amplitude following glutamate exposure. The inclusion of an endothelial layer mimicking the blood brain barrier enabled drug screening under physiologically-relevant conditions, where co-treatment with rapamycin and bosutinib partially restored contractile function.

While these 3D systems represent a major step forward, their full potential is realized when combined with biofabrication technologies that enable precise control over tissue architecture. In this context, 3D bioprinting has emerged as a powerful platform for engineering neural microenvironments with defined spatial organization and tunable biophysical properties. Unlike self-organized organoid systems, bioprinting enables deterministic placement of cells and materials, allowing the reconstruction of disease relevant niches with controlled geometry and reproducibility.

Bioprinting technologies have enabled the fabrication of spatially organized neuromuscular tissues, where motor neurons, glial cells, and muscle fibers can be positioned in defined architectures. Work by Kim et al. demonstrated EBB of aligned skeletal muscle constructs integrated with neural cells, resulting in robust neuromuscular junction formation and functional recovery in vivo (Kim et al., 2020). The optimization of bioinks, including fibrinogen, gelatin, HA, and glycerol-based formulations, was essential for achieving appropriate mechanical properties and supporting cellular organization.

Similarly, bioprinted scaffolds have been used to guide the differentiation and maturation of patient-derived motor neurons. Studies employing gelatin laminin bioinks and mesoporous silica particles have demonstrated that matrix stiffness and biochemical cues critically regulate neurite extension and neuronal network formation (Han et al., 2025). Softer hydrogel environments promote enhanced cell viability and connectivity, highlighting the importance of tuning ECM properties to mimic the native neural microenvironment. Complementary work using patient-derived cells embedded in bioengineered matrices further confirms that 3D environments support electrophysiologically active motor neurons suitable for modeling ALS pathology (Scarian et al., 2022).

A defining advantage of bioprinted systems lies in their ability to integrate multiple cell types in spatially controlled configurations, enabling the study of non-cell autonomous degeneration within engineered microenvironments. Engineered co-culture systems consistently demonstrate superior neuromuscular junction maturation compared to simplified cultures. For example, fibrin-based 3D neuromuscular models exhibit enhanced muscle fiber alignment, adult myosin expression, and functional innervation, all of which are essential for accurately modeling ALS related degeneration (Bakooshli et al., 2019). Similarly, bioengineered motor unit models incorporating iPSC-derived neurons and primary muscle tissues reveal improved acetylcholine receptor clustering and contractile functionality in 3D settings (Rimington et al., 2021).

Beyond structural organization, bioprinted neural microenvironments enable precise control over key physical and biochemical parameters that govern disease progression. Mechanical stiffness, matrix viscoelasticity, and anisotropy can be tuned to influence neuronal maturation and synaptic stability. Gradients in oxygen tension and nutrient diffusion can be engineered to model hypoxia driven degeneration and metabolic stress, which are increasingly recognized as contributors to ALS pathology. Furthermore, the ability to control temporal dynamics allows the investigation of disease progression from early synaptic dysfunction to advanced neurodegeneration within a single platform.

Despite these advances, several challenges remain. Many current models lack full integration of immune components, which play a critical role in ALS progression. Scalability and standardization also remain limiting factors for high-throughput drug screening applications (Rouleau et al., 2023). Nevertheless, 3D bioprinted neural microenvironments represent a significant step toward predictive and translational disease models. By enabling precise control over cellular organization, matrix composition, and microenvironmental cues, these systems bridge the gap between reductionist in vitro models and complex in vivo physiology (Islam et al., 2024; Rouleau et al., 2023).

Collectively, the convergence of stem cell biology, biomaterials science, and bioprinting technologies is redefining how neurodegenerative diseases are modeled. Rather than merely increasing biological complexity, the next generation of ALS models is defined by the ability to engineer and control that complexity, positioning bioprinted neural microenvironments as programmable platforms for mechanistic discovery, drug development, and personalized medicine (Islam et al., 2024).

### 5.4 Alzheimer's disease (AD) models

Within NMDC framework, AD represents one of the most concurrent disruption of defined codes. AD, accounting for approximately 50–70% of all dementia cases, is a progressive neurodegenerative disorder characterized by cognitive decline, memory impairment, and behavioral dysfunction. Neuropathologically, AD is defined by extracellular accumulation of amyloid-β (Aβ) plaques and intracellular aggregation of hyperphosphorylated tau in neurofibrillary tangles, leading to synaptic dysfunction, neuronal loss, and progressive brain atrophy (Deture and Dickson, 2019; Selkoe and Hardy, 2016). These pathologies can be categorized under NMDC, with mechanical code being disrupted via plaque-associated stiffness changes, and biochemical code being disrupted with protein accumulation. While familial AD cases are linked to mutations in APP and presenilin genes, the majority of cases are sporadic and arise from complex interactions between genetic and environmental factors, with APOE4 representing the strongest genetic risk factor (Karch et al., 2014). Beyond neuronal pathology, AD is increasingly recognized as a multi-cellular disease involving neuroinflammation and neurovascular dysfunction, indicating the disruption of immune/barrier code. Chronic activation of microglia and astrocytes drives sustained inflammatory signaling while BBB disruption alters vascular integrity and immune responses (Heneka et al., 2024; Sweeney et al., 2018).

Despite extensive use of transgenic animal models, species-specific differences limit their ability to fully recapitulate human AD pathology, particularly sporadic and age-dependent phenotypes (Drummond and Wisniewski, 2017; Alexandra Lopes et al., 2025). Similarly, 2D in vitro systems capture isolated molecular mechanisms but lack the spatial organization, cellular diversity, and microenvironmental complexity required for physiologically relevant modelling (Centeno et al., 2018). These limitations have driven the development of 3D human-relevant platforms, including bioprinted constructs and brain organoids. Essential microenvironmental features for modeling AD include a multi-cellular system with neurons, astrocytes, microglia, and endothelial cells, the spatial organization of synaptic and inflammatory niches, ECM-mediated regulation of Aβ aggregation and propagation, and the incorporation of BBB dysfunction to capture neurovascular contributions. Correspondingly, key engineering targets include the reconstruction of multi-cellular architecture to recapitulate responses to Aβ toxicity and tau pathology, alongside the integration of neuroimmune crosstalk, neurovascular interfaces, and BBB function. Furthermore, the generation of defined protein aggregation niches within controlled biochemical and biophysical environments is critical for modeling disease progression.

Recent advances in 3D bioprinting enable controlled reconstruction of neural microenvironments with defined architecture and function. For example, coaxial bioprinting of APP-mutant neural progenitor cells supports the formation of self-organizing neural networks with enhanced AD-like pathological features compared to 2D systems (Zhang et al., 2021). Similarly, microfluidic bioprinting approaches using patient-derived iPSCs allow generation of constructs enriched in vulnerable neuronal populations, such as basal forebrain cholinergic neurons, while enabling incorporation of smart bioinks for controlled delivery of biochemical cues (Benwood et al., 2023).

Bioprinted systems further enable investigation of neurogenesis impairment and synaptic dysfunction. Constructs incorporating extracellular Aβ oligomers demonstrate reduced cell viability, increased oxidative stress, and progressive gene expression changes, highlighting the role of pathological microenvironments in stem cell dysfunction (Ferreira et al., 2024). Similarly, spatially organized forebrain models reveal reduced neurite complexity and impaired connectivity, directly linking 3D tissue architecture to functional deficits (Fig. 5A) (Whitehouse et al., 2025).

**Fig. 5:**
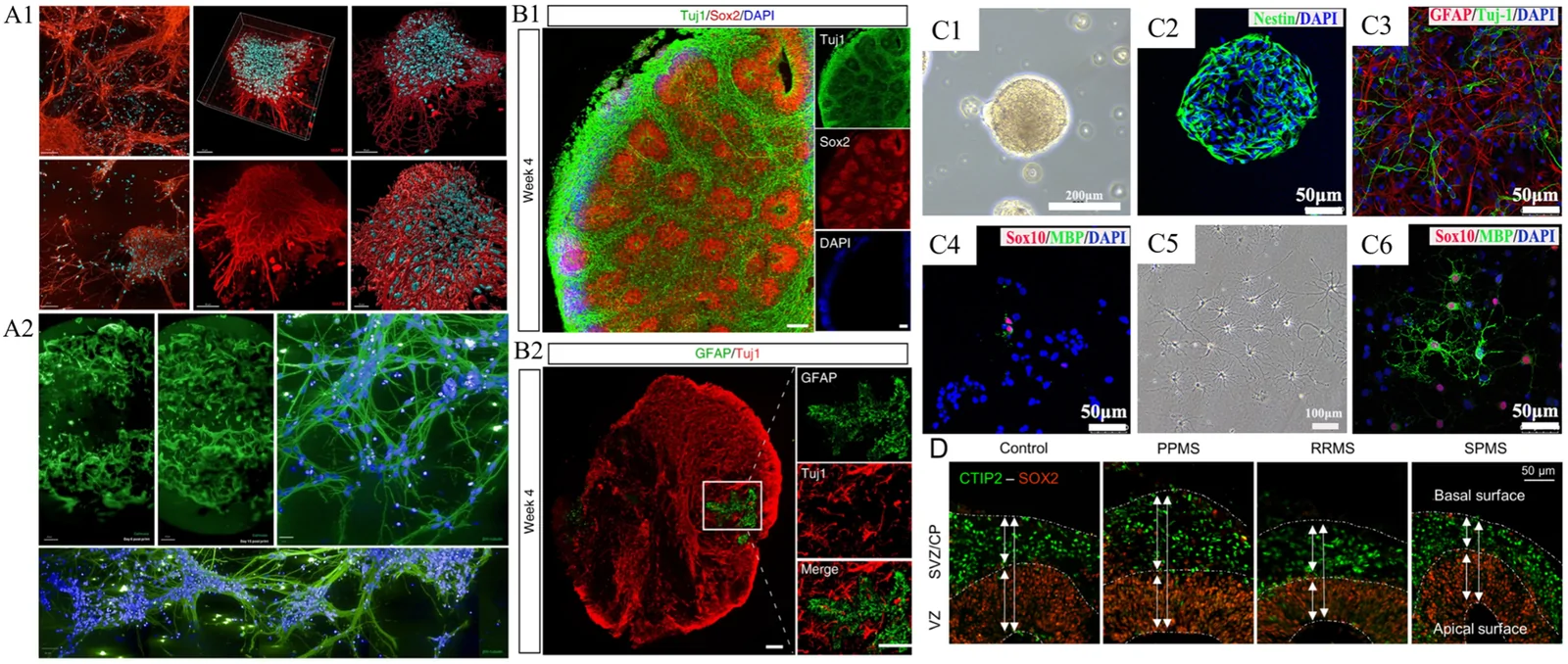
AD Models (A-B). (A1-2) Quantification of neural connectivity in a 3D bioprinted tri-matrix model: (A1) MAP2+ neurite extensions (red) within a hydrogel matrix; (A2) whole-well scans and high-magnification images of βIII-tubulin (green) neurite outgrowth at Day 6 nd Day 15 post-bioprinting. (B1-2) Generation and characterization of patient iPSC-derived cerebral organoids: (B1) representative images of the ventricular zone (VZ)-like structure formed by new-born neurons (Tuj1, green) and neural progenitor cells (Sox2, red) in cerebral organoids at week 4 of differentiation; (B2) proliferation and migration of astrocytes within cerebral organoids; the differentiation pattern of astrocytes in organoids were monitored by GFAP immunostaining (astrocytic marker) at week 4 representative image of the ventricular zone (VZ)-like structure containing newborn neurons (Tuj1, green) and progenitor cells (Sox2, red) at week 4; (E, F) proliferation and migration of GFAP+ astrocytes (red) at week 4. Representative images of the ventricular zone (VZ)-like structure formed by new-born neurons (Tuj1, green) and neural progenitor cells (Sox2, red) in cerebral organoids at week 4 of differentiation. e, f Proliferation and migration of astrocytes within cerebral organoids; the differentiation pattern of astrocytes in organoids were monitored by GFAP immunostaining (astrocytic marker) at week 4 (e) and 12 (f), respectively. MS Related Models (C-D). (C) Identification of NSCs and oligodendrocytes (OLGs) for 3D-bioprinted regenerative scaffolds: (C1) NSCs forming neurospheres; (C1) NSCs positively expressing nestin protein; (C3, C4) verification of differentiation potential with Tuj-1 (neurons), GFAP (astrocytes), SOX10 (OLGs) and MBP (mature OLGs) antibodies; (C5) mature OLGs extending multiple processes; (C6) co-expression of MBP and Sox10 of OLGs. (D) Structural organization of human cerebral organoids at Day 42: immunofluorescence shows the distribution of SOX2+ stem cells and CTIP2+ neurons, highlighting the clear separation between the ventricular zone (VZ) and the cortical plate (CP) used to study innate differentiation defects in MS types: PPMS, RRMS and SPMS. Panel A adapted from (Whitehouse et al., 2025), B from (Zhao et al., 2020), C from (Liu et al., 2022), D from (Daviaud et al., 2023). All licensed under CC BY.

Beyond disease modeling, bioprinting approaches are expanding toward therapeutic applications. Engineered brain-like constructs with anatomically optimized geometries have demonstrated improved integration and engraftment in vivo, suggesting potential for regenerative strategies (Gai et al., 2026). In parallel, incorporation of nanomaterials into engineered systems has been shown to reduce Aβ-induced oxidative stress and inflammation, providing additional avenues for model development and intervention (Chiang et al., 2021).

Despite these advances, bioprinted constructs are constrained by predefined architectures and may not fully capture intrinsic developmental programs. In contrast, organoid systems rely on self-organization to generate diverse neuronal subtypes and early network formation, making them particularly suitable for modeling early disease processes. Patient-derived organoids capture genotype-specific effects, including APOE4 (apolipoprotein E4)-driven increases in tau pathology, synaptic loss, and neuronal degeneration (Fig. 5B) (Zhao et al., 2020). Organoid models also enable investigation of environmental contributions to disease. For example, serum-exposed brain organoids mimic blood–brain barrier disruption and induce AD-like phenotypes, including Aβ accumulation, tau phosphorylation, and neuroinflammatory responses (Chen et al., 2021). Similarly, familial AD organoids recapitulate early network dysfunction, including dysregulated calcium signaling and increased neuronal activity, while simplified systems reproduce core pathological features such as amyloid aggregation and apoptosis (Yin and Vandongen, 2020; Choe et al., 2024).

To further increase physiological relevance, multi-cellular organoid systems incorporating immune and vascular components have been developed. Neuroimmune and vascularized organoids exposed to patient-derived pathological seeds exhibit hallmark features including plaque formation, neuroinflammation, synaptic loss, and neuronal degeneration (Ji et al., 2025). Similarly, assembloid models integrating region-specific brain tissues and immune elements enable investigation of intercellular interactions and disease propagation (Becerra-Calixto et al., 2025).

Despite these advances, organoid systems lack precise control over spatial organization and microenvironmental parameters. In contrast, 3D bioprinting enables deterministic control over cellular architecture, ECM composition, and mechanical properties, allowing the engineering of defined pathological niches. To develop physiologically-relevant AD models, future systems must integrate key design principles reflecting disease complexity. Effective models should incorporate neurons, astrocytes, microglia, and vascular components within spatially organized architectures. Matrix composition and mechanical properties must be tuned to reflect native brain tissue, as these parameters regulate neuronal connectivity, glial activation, and disease progression. In addition, controlled incorporation of pathological cues, including Aβ and tau, alongside inflammatory and vascular components, is essential for modeling disease initiation and progression. Spatial organization is particularly critical, as synaptic networks, neuroinflammatory regions, and vascular interfaces interact dynamically within the diseased brain. Integration of transport dynamics and BBB mimetic systems will further enhance physiological relevance. Altogether, successful AD models must move beyond reproducing individual pathological features toward engineering the microenvironmental conditions that govern disease progression. The convergence of bioprinting, organoid technologies, and multi-cellular integration strategies will enable next-generation platforms capable of capturing the complexity of AD while supporting translational applications in drug discovery and personalized medicine.

### 5.5 MS models

MS is a chronic autoimmune and neurodegenerative disorder of the central nervous system, characterized by focal demyelination, axonal degeneration, and progressive neurological disability (Filippi et al., 2018). Although clinical subtypes reflect heterogeneous disease trajectories, a unifying feature of MS pathology is the failure of effective remyelination within demyelinated lesions (Andersson et al., 1999). In NMDC terms, it is mainly an immune/barrier and biochemical code disorder, in which BBB breakdown, ECM remodeling and chronic glial inflammation collectively disrupt remyelination.

Oligodendrocyte precursor cells (OPCs) are initially recruited to sites of injury and contribute to myelin repair; however, this process becomes progressively impaired due to both intrinsic cellular dysfunction and extrinsic inhibitory signals within the lesion microenvironment (Levine et al., 2001). In this context, MS represents a fundamentally microenvironment-driven disease in which remyelination failure is dictated not only by cellular deficits but by extracellular matrix composition, inflammatory signaling, and spatial tissue organization. Neuroinflammation further amplifies disease progression, with chronic active lesions sustained by interactions between microglia, astrocytes, and infiltrating immune cells (Kuhlmann et al., 2023).

To develop physiologically relevant MS models, future systems must integrate key design principles that reflect lesion microenvironment complexity. Recapitulate these pathological features in a model requires a complex microenvironment involving coordinated interactions between neurons, oligodendrocyte lineage cells, astrocytes, and microglia, alongside infiltrating peripheral immune cells. In addition, ECM remodeling within the lesion environment and BBB dysfunction enabling immune infiltration are the engineering targets that are critical in shaping disease progression.

Despite advances in understanding these mechanisms, conventional in vivo and 2D in vitro models fail to capture the spatial and multi-cellular complexity of MS pathology, limiting their ability to model disease progression and therapeutic response. 3D bioengineered systems address this gap by enabling controlled reconstruction of neural microenvironments. Although fully integrated MS-specific bioprinted models remain limited, emerging studies highlight key design principles underlying demyelination and remyelination.

Biomimetic artificial axons fabricated through micro-scale bioprinting support OPC adhesion and myelination, demonstrating that axonal geometry and mechanical properties directly regulate oligodendrocyte behavior (Espinosa-Hoyos et al., 2018; Jagielska et al., 2023). Similarly, spatially organized constructs incorporating oligodendrocyte lineage cells show that remyelination is strongly influenced by multi-cellular interactions and ECM composition (Liu et al., 2022; Joung et al., 2018). Complementary engineered systems further demonstrate that ECM composition, including HA-rich environments, regulates glial behavior and can inhibit oligodendrocyte maturation (Schroyer et al., 2025; Tang et al., 2020).

Astrocyte–matrix interactions and immune responses can be modulated using tunable biomaterials, where hydrogel composition and scaffold architecture regulate astrocyte activation and macrophage polarization (Matthiesen et al., 2023; Dutta et al., 2023; Martinelli et al., 2025). These findings highlight that remyelination outcomes are governed by the balance between inflammatory and regenerative microenvironmental states.

In parallel, organoid and assembloid systems capture intrinsic cellular programs and patient-specific phenotypes. Patient-derived cerebral organoids reveal impaired oligodendrocyte maturation and reduced neural stem cell populations, while inflammatory stimulation induces disease-relevant phenotypes that can be partially reversed therapeutically (Daviaud et al., 2023; Sadiq et al., 2026; Fagiani et al., 2025). Advanced assembloid models incorporating immune and vascular components further enable controlled investigation of neuroimmune interactions (Fagiani et al., 2024).

Despite these advances, organoid systems lack precise control over spatial organization and microenvironmental parameters. In contrast, 3D bioprinting enables deterministic control over cellular architecture, ECM composition, and mechanical properties, providing a unique platform for engineering lesion-like environments with defined geometry (Datta et al., 2017; Peng et al., 2017).

Controlled inflammatory gradients are essential for replicating chronic lesion environments, enabling simultaneous study of degeneration and repair. Spatial organization remains a defining requirement, as MS lesions exhibit distinct regions of demyelination, inflammation, and gliosis that cannot be reproduced in homogeneous systems. The ability to engineer such architectures through bioprinting represents a critical advantage in modeling disease progression. Integration of vascular and transport components, including BBB mimetic systems, will further enhance physiological relevance.

Collectively, successful MS models must move beyond reproducing cellular composition toward engineering the microenvironmental constraints that govern disease progression. The convergence of bioprinting, organoid technologies, and immune integration strategies will enable next-generation platforms capable of capturing the dynamic and heterogeneous nature of MS pathology while supporting translational applications in drug discovery and personalized medicine (Table 1).

**Table 1:** Engineering design principles for 3D bioprinted neural microenvironments in neurodegenerative diseases.

**Disease**	**Core Pathology**	**Microenvironment Drivers**	**Engineering Targets**	**Functional Readouts**	**Model Type**
PD	Dopaminergic neuron loss, α-syn aggregation	Neuroinflammation, oxidative stress, neuron–glia interaction	Midbrain constructs, dopaminergic support, stress gradients	Dopamine release, neuron survival, α-syn levels	Organoids, microfluidic systems (Zuccoli et al., 2025; Kim et al., 2025; Gerasimova et al., 2025; Mao et al., 2024; Chlebanowska et al., 2020; Joshi et al., 2025; Raja et al., 2022)
MG	NMJ disruption, AChR targeting	Synaptic alignment, receptor clustering, immune activity	Aligned neuron–muscle constructs, immune-integrated NMJ	Muscle contraction, Ca²⁺ signaling, synaptic activity	Bioengineered NMJ models, organoids, co-culture systems (Rimington et al., 2021; Shin et al., 2025; Vila et al., 2021)
ALS	Motor neuron degeneration, NMJ failure	Glial toxicity, ECM influence, neuroinflammation	Motor neuron–muscle interfaces, glia integration	NMJ function, neuron survival, TDP-43 aggregation	Co-culture systems, assembloids, optogenetic models (Wasielewska et al., 2024; Massih et al., 2023; Rimington et al., 2021; Kim et al., 2020; Baofengfeng et al., 2022; Zondler et al., 2016; Han et al., 2025; Scarian et al., 2022; Bakooshli et al., 2019)
AD	Aβ plaques, tau pathology	Neuroinflammation, BBB dysfunction, ECM remodeling	Multi-cellular constructs, neurovascular modeling	Synaptic activity, Aβ/tau levels, connectivity	Organoids, vascularized systems (Whitehouse et al., 2025; Zhao et al., 2020; Zhang et al., 2021; Ferreira et al., 2024; Gai et al., 2026; Chen et al., 2021; Yin and Vandongen, 2020; Choe et al., 2024; Ji et al., 2025; Becerra-Calixto et al., 2025; Benwood et al., 2023)
MS	Demyelination, remyelination failure	Immune infiltration, ECM inhibition, lesion heterogeneity	OPC integration, axon-mimetic scaffolds, gradient systems	Myelination, OPC maturation, inflammation	Organoids, biomaterial models (Liu et al., 2022; Daviaud et al., 2023; Jagielska et al., 2023; Joung et al., 2018; Schroyer et al., 2025; Tang et al., 2020; Sadiq et al., 2026; Fagiani et al., 2025; Fagiani et al., 2024; Espinosa-Hoyos et al., 2018)

## 6 Organoids, neurovascular and neuroimmune integration

Organoid-based and bioprinted neural models represent complementary strategies for neural tissue engineering, rather than being mutually exclusive model categories. Organoids mainly rely on stem cell driven self organization, allowing developmental programs to generate native like tissue with relatively limited external spatial control. Assembloids extend this principle by combining different tissue domains, in which a controlled property is added while self-organizing is kept. In contrast, bioprinting rely more strongly on externally controlled organization and deliberate positioning of cells, biomaterials along with NMDC explained heavily through this review. Therefore, these approaches occupy different positions along the self organization to engineered construct continuum, with hybrid strategies combining intrinsic tissue development and self organization with external microenvironment control. This section explains organoid and assembloid parts of this continuum in depth.

Brain organoids have emerged as powerful platforms for modeling human neurodevelopment and neurological diseases. However, their physiological relevance remains limited by the absence of key non-neuronal components, particularly vascular and immune elements (Tan et al., 2023; Urrestizala-Arenaza et al., 2024). In vivo, brain function is governed not only by neuronal networks but also by tightly coupled interactions between neural, immune, and vascular systems (Norris and Kipnis, 2019; Zhong et al., 2025). These interactions regulate tissue homeostasis, synaptic organization, and response to injury, highlighting that neuron-centric models are inherently insufficient to capture the complexity of the human brain. As a result, next-generation in vitro systems must move toward integrated, multi-system platforms that better reflect the dynamic microenvironment of neural tissues (Han et al., 2025).

Early brain organoid models successfully recapitulated aspects of human brain development, including progenitor zone formation and early neuronal differentiation. However, unguided differentiation approaches often resulted in heterogeneous tissues with mixed regional identities, limiting reproducibility and disease-specific modeling (Lancaster and Knoblich, 2014). The development of guided differentiation protocols, including Dual Small Mothers Against Decapentaplegic (dual-SMAD) inhibition and controlled morphogen signaling, enabled the generation of region-specific organoids representing cortical, midbrain, and hippocampal structures (Scuderi et al., 2025; Zhang et al., 2022). More recently, the fusion of region-specific organoids into assembloids has provided a platform to study inter-regional connectivity and circuit-level interactions (Birey et al., 2017; Xiang et al., 2019; Andersen et al., 2020).

Despite these advances, brain organoids still exhibit critical limitations, including hypoxia-induced necrotic core formation, limited nutrient diffusion, incomplete cellular maturation, and batch-to-batch variability. These limitations arise from two partially different sources: intrinsic variability associated with self organizing development and engineering constraints such as absence of controlled transport, vascularization, and spatial patterning. Bioprinting and related biofabrication advances can address the latter source by imposing defined geometry, cellular positioning, and perfusion, while potentially limiting intrinsic genetic program. Vascularization strategies have partially addressed these issues by improving oxygen and nutrient delivery, reducing cell death, and enhancing neuronal maturation (Tan et al., 2023). In parallel, the incorporation of engineered biomaterials has improved reproducibility by providing defined mechanical and biochemical cues during organoid development (McMurtrey, 2016; Muñiz et al., 2023). However, these approaches alone remain insufficient to fully capture the complexity of in vivo brain physiology, particularly in the absence of immune and vascular interactions.

To overcome these limitations, the integration of neuroimmune and neurovascular components has emerged as a key direction in advanced brain modeling. These elements are not only essential for enhancing structural and functional realism but also for capturing disease-relevant processes such as inflammation, barrier dysfunction, and intercellular signaling that are central to many neurological disorders.

### 6.1 Neuroimmune integration in organoids

Neuroimmune interactions represent a critical yet historically underrepresented component of brain organoid systems. Conventional organoids derived from pluripotent stem cells primarily consist of neuroectodermal lineages, including neurons, astrocytes, and oligodendrocytes, but lack mesoderm-derived immune populations such as microglia (Norris and Kipnis, 2019). This absence significantly limits the ability of organoids to model processes regulated by immune–neural interactions, including synaptic pruning, inflammatory signaling, and cytokine-mediated neuromodulation (Tian et al., 2012).

Microglia, the resident immune cells of the central nervous system, originate from yolk sac-derived progenitors and play essential roles in brain development and homeostasis (Gomez Perdiguero et al., 2014). They regulate synaptic refinement through phagocytosis, clear apoptotic debris, and modulate neuronal activity through cytokine secretion (Norris and Kipnis, 2019). Although it has been shown that the microglia can innately develop in a cerebral organoid, generally the dual-SMAD inhibition process in organoid formation prevents this development (Ormel et al., 2018). To address their absence, several strategies have been developed, including co-culture with iPSC-derived microglia and the incorporation of mesodermal progenitors during early differentiation (Hong et al., 2023; Fagerlund et al., 2021). These approaches have demonstrated that microglia can integrate into organoid tissue, migrate throughout the construct, and adopt functional phenotypes resembling in vivo states (Fagerlund et al., 2021).

Cytokines act as key mediators linking immune activity to neuronal function and network maturation. Molecules such as IL-6, TNF-α, and TGF-β regulate neuronal excitability, synaptic plasticity, and glial activation states (Kennedy and Silver, 2022). In organoid systems, modulation of cytokine signaling enables the modeling of disease-relevant conditions, including chronic inflammation and neurodegenerative processes (Yuan et al., 2025). Furthermore, cytokine-driven activation of astrocytes can induce reactive phenotypes that alter metabolic support and disrupt neural network function, emphasizing the role of immune signaling in shaping neural dynamics (Zipp et al., 2023).

Beyond microglia, infiltrating immune cells such as T-cells and macrophages play critical roles in central nervous system pathology, particularly in neurodegenerative and autoimmune diseases. Recent advances have enabled the incorporation of peripheral immune cells into organoid systems through co-culture approaches and microfluidic platforms that mimic immune cell infiltration (Gerasimova et al., 2025; Liang et al., 2026). These hybrid models enable the study of bidirectional communication between the central nervous system and peripheral immune system, providing insights into neuroinflammation, immune-mediated neurodegeneration, and disease progression.

These interactions are particularly critical in the context of neurodegenerative diseases, where the interplay between neural, immune, and vascular systems is a key driver of pathology. In AD, microglia-mediated neuroinflammation, dysregulated cytokine signaling, and BBB disruption contributes to amyloid accumulation and progressive neuronal loss (Heneka et al., 2024). In PD, chronic immune activation and vascular dysfunction have been associated with dopaminergic neuron degeneration and altered neural network activity (Tansey et al., 2022). Similarly, in MS, infiltration of peripheral immune cells and loss of neurovascular integrity drive demyelination and neurodegeneration (Woo et al., 2024). These examples demonstrate that neuroimmune interactions are not secondary features but central mechanisms underlying disease progression.

### 6.2 Neurovascular integration in organoids

In vivo, brain function is tightly coupled to the neurovascular unit, a highly coordinated system composed of endothelial cells, pericytes, astrocytes, and neurons. These components collectively regulate cerebral blood flow, metabolic exchange, and the integrity of the BBB, which is essential for maintaining a stable and controlled neural microenvironment (Zlokovic, 2008; Zhong et al., 2025).

Conventional brain organoids lack functional vasculature, resulting in limited oxygen and nutrient diffusion, hypoxia, and increased cell death, particularly in core regions.

To address these challenges, multiple strategies have been developed to introduce vascular features into organoid systems. These include co-culture with ECs, incorporation of vascular progenitors, and the use of microfluidic platforms and engineered scaffolds to promote the formation of perfusable vascular networks (Tan et al., 2023; Pham et al., 2018; Cakir et al., 2019). Vascularized organoids have demonstrated improved viability, reduced necrotic core formation, and enhanced neuronal maturation compared to non-vascularized models.

Importantly, the integration of vascular elements enables the modeling of BBB function, including selective permeability and transport dynamics (Vatine et al., 2019). This is particularly relevant for studying neurodegenerative diseases, stroke, and therapeutic delivery, where BBB dysfunction plays a central role in disease progression and treatment response (Bergmann et al., 2018).

#### 6.2.1 Vascularization strategies

Prominent efforts to model the neurovascular interface, particularly BBB, relies on simplified systems that mimic the flow and selective permeability, such as microfluidic devices, hydrogels, and transwell-based platforms (Jeong et al., 2018; Singh et al., 2023; Stone et al., 2019). While these approaches enable the investigation of endothelial-neural cell interactions, they especially fail to fully explain developmental dynamics and the functional complexity of the BBB (Aazmi et al., 2022). BBB organoids generated through co-culture of ECs, pericytes, and astrocytes in low-attachment conditions have shown improved physiological relevance. These models exhibit important BBB features such as tight junction formation and drug efflux pumps and have proven more effective than the transwell system (Bergmann et al., 2018; Cho et al., 2017).

In addition to the advances in BBB organoids, several strategies have been developed to introduce vascular features directly into brain organoids. One approach is the co-culture or fusion of neural organoids with ECs or vascular progenitors (Pham et al., 2018). ECs such as human umbilical vein endothelial cells (HUVECs), human brain microvascular endothelial cells (HBMVECs), or iPSC-derived ECs, have been explored for this purpose (Shi et al., 2020; Navarro et al., 2025). ECs can self-organize to form vascular like networks withing the organoid that serve as a primitive vascularization. Studies have shown the presence of lumen-containing structures inside the brain organoids when co-cultured with HUVECs. These vascularized organoids have reduced hypoxia, decreased apoptosis, and improved neuronal differentiation compared to non-vascularized controls (Shi et al., 2020). Similarly, fusion of vascular spheroids with brain organoids has been used to generate hybrid systems in which the vascular component infiltrates the neural tissue (Kook et al., 2022). These fused systems can include not only vascular cells but also microglia, allowing modeling of neuroimmune–vascular interactions, which will be explained in detail in the next section.

Another important strategy is the genetic induction of EC identity within organoids. Notable studies utilized the transient overexpression of the transcription factor ETV2 or signaling molecules such as VEGF in IPSCs, driving the differentiation of endothelial like cells within developing brain organoids (Cakir et al., 2019; Skylar-Scott et al., 2022). These induced ECs self-organize into complex vascular like networks like co-cultured organoids, and again display key features of early vasculature, such as lumen formation and expression of EC markers. This approach is significant due to the generation of vascular components intrinsically within the organoid, creating a more integrated and developmentally relevant system.

Transplantation-based approaches have also provided valuable insights into organoid vascularization. In one study, human brain organoids transplanted into the mouse brain became infiltrated by host vasculature, resulting in improved survival, enhanced neuronal maturation, and functional synaptic activity (Mansour et al., 2018). The host-derived blood vessels not only supplied oxygen and nutrients but also contributed to the establishment of a more physiologically-relevant microenvironment. While this method relies on in vivo systems and is therefore less controllable than in vitro approaches, it demonstrates the impact of vascularization on organoid development and highlights the importance of perfusion.

Lastly, bioengineering approaches in parallel with organoid technology advances have been powerful vascularization strategies in recent years. Advanced techniques such as 3D bioprinting and organ-on-chip platforms aim to create perfusable vascular networks (Jeong et al., 2018; Zhu et al., 2021; Cai et al., 2025; Salmon et al., 2022). These systems offer to introduce flow dynamics, enable controlled perfusion, the intensity of shear stress, and precise regulation of the biochemical microenvironment, all of which are critical factors in vascular development and function. For example, brain organoids cultured in microfluidic devices with continuous media flow exhibit improved nutrient delivery, reduced hypoxia, and enhanced neuronal maturation (Cho et al., 2021). In addition, co-culture of ECs, pericytes, and neural progenitors within microfluidic systems has been shown to generate organized vascular networks that closely interact with neural tissue (Salmon et al., 2022). These platforms can also mimic aspects of blood flow and BBB dynamics, providing a more physiologically relevant environment compared to static cultures.

Despite these advances, challenges remain in fully mimicking the complexity of the in vivo neurovascular system. Current vascularized organoids often lack fully functional perfusable vessels and may not achieve the hierarchical organization of the brain vasculature. Additionally, the integration of all NVU components, including pericytes and astrocytes in appropriate spatial positions, remains an ongoing challenge.

#### 6.2.2 Role of physical cues

The physical microenvironment plays a crucial role in the successful integration and maturation of vascular components. Shear stress, generated by fluid flow, is a key regulator of EC behavior, promoting alignment, maturation, and barrier formation. In the absence of flow, ECs often fail to fully mature and may not establish functional barrier properties (Cucullo et al., 2011). Similarly, the composition and mechanical properties of the ECM influence vascular organization and stability, while also influencing vascular and glial behaviour (Reed et al., 2019). The use of defined biomaterials, such as synthetic hydrogels or dECM, has been shown to support vascular network formation and improve overall organoid architecture (Cho et al., 2021; Barry et al., 2017). These materials provide not only structural support but also biochemical cues that guide cell differentiation and interaction. In vitro models lacking these physical and biochemical cues from the environment often fail to replicate NVU and BBB properties, highlighting the importance of mechanical factors in neurovascular integration.

### 6.3 Neuroimmune–neurovascular interactions

While the separate incorporation of immune and vascular components has significantly improved brain organoid models, the goal is to reconstruct the crosstalk of them as in vivo. In vivo, neural, immune, and vascular systems do not function in isolation but instead form a highly dynamic and interdependent network that regulates brain homeostasis, development, and response to environmental stimuli (Muoio et al., 2014; Dantzer, 2017; Huang et al., 2020). The neurovascular unit and resident immune populations are tightly coupled through bidirectional signaling pathways that influence barrier function, cellular recruitment, and neural activity. Therefore, capturing these interactions within organoid systems is very important for achieving physiologically relevant models. Recent advances in organoid engineering aim to fully capture this complex dynamic, combining vascularized and immune-competent systems. These integrated models allow the study of how immune signaling influences vascular behavior and, conversely, how vascular structures regulate immune cell function and distribution.

#### 6.3.1 Microglia–EC crosstalk

Microglia–endothelial interactions are a central component of neuroimmune–neurovascular communication. Their interactions during development are mediated by signaling crosstalk and contribute to the coordinated formation and maturation of the neurovascular and neuroimmune systems . Microglia contribute to vascular development by secreting a variety of cytokines and signaling molecules that directly influence EC behavior, including proliferation, barrier integrity, and inflammatory responses (Ronaldson and Davis, 2020). For example, microglial cytokines can enhance or disrupt tight junction protein expression (claudin-5, occludin, ZO-1), thereby dynamically regulating barrier permeability (Yu et al., 2015; Cucullo et al., 2011). Pro-inflammatory cytokines such as IL-1β and TNF-α have been shown to transiently increase permeability in vitro, while TGF-β signaling supports junctional stability and maintains selective permeability (Ronaldson and Davis, 2020). In organoid systems incorporating microglia, such as a fused system of cerebral and vascular organoids containing microglia-like cells, similar signaling pathways have been observed, where activated microglia alter the local micro-environment, respond to immune stimuli, and impact endothelial-like structures (Han et al., 2025; Sun et al., 2022).

Conversely, ECs regulate immune signaling by controlling the entry and distribution of cytokines and circulating factors through BBB-like properties. In vascularized organoids exhibiting tight junction formation and selective permeability, endothelial layers act as regulators of molecular exchange, thereby influencing the immune microenvironment (Cakir et al., 2019). Although these barrier properties are less mature than in vivo BBB, they are sufficient to model important aspects of molecular transport and tissue identity.

In addition, ECs produce signaling molecules such as chemokines and cytokines, that influence microglial migration and activation states. This bidirectional communication creates a feedback loop in which immune activation can alter vascular function, and vascular-derived signals can, in turn, regulate immune responses (Haruwaka et al., 2019). Reconstructing this complex crosstalk in organoids provides a more accurate representation of in vivo, particularly in neurodegenerative conditions, where disruption of barrier integrity and dysregulated immune activation are closely related.

#### 6.3.2 Astrocytes as intermediaries

Astrocytes serve as a bridge between neuroimmune and neurovascular interactions. Their endfeet wrap around the blood vessels and contribute to BBB maintenance, while their soma and processes also interact with neurons and microglia (Liu et al., 2020). Astrocytes secrete a range of trophic factors and cytokines, including VEGF, IL-6, and CXCL1, which also influence vascular stability and immune responses (Liu et al., 2020; Lawrence et al., 2023). In organoids, astrocytic incorporation enhances both neural network activity and neural maturation (Zheng et al., 2025). Astrocytes can modulate microglial activation states, promoting either pro-inflammatory or anti-inflammatory phenotypes depending on the local microenvironment (Kim and Son, 2021). Additionally, astrocytes respond to vascular signals by adjusting their metabolic support to neurons and they are modulated by VEGF signaling (Stobart and Anderson, 2013; Kou et al., 2019).

This intermediary role of astrocytes is especially critical in modeling pathological conditions. For example, in a vascularized neuroimmune model containing neurons, astrocytes, microglia and vessels, elevated levels of cytokines and microglial activation have been observed in a neurodegenerative disease context (Ji et al., 2025). The inclusion of astrocytes in organoid models, therefore, allows for the high physiological relevance of modelling the interactions between immune cells, vasculature, and neurons, which are essential for understanding the developmental and pathological processes.

#### 6.3.3 Functional outcomes

The most significant effect of the integration of neuroimmune and neurovascular components within brain organoids is the coupled regulation of barrier function and immune signaling. Cytokine-mediated modulation of ECs influences tight junction stability and permeability, while vascular structures regulate the distribution and concentration of signaling molecules within the tissue (Nzou et al., 2020).

Another key outcome is the impact on neuronal activity and network formation. Immune signaling molecules, particularly cytokines, can directly influence neuronal excitability and synaptic plasticity, while vascular components provide metabolic support necessary for sustained neural activity (Mansour et al., 2018; Cakir et al., 2019). In integrated organoid systems, these combined effects result in more synchronized and functionally mature neural networks. This is particularly relevant in neurodegenerative diseases such as AD, where pro-inflammatory cytokines disrupt synaptic function and contribute to network hyperexcitability, and in PD, where neuroinflammation and impaired metabolic support contribute to dopaminergic neuronal dysfunction (Ji et al., 2025; Elvira et al., 2025).

Additionally, neuroimmune–neurovascular interactions contribute to cellular organization and structural maturation. The presence of vascular networks reduces hypoxia and supports long-term viability, while immune cells regulate processes such as synaptic pruning and debris clearance (Tan et al., 2023; Sun et al., 2022). Together, these interactions promote a more stable and organized tissue architecture compared to conventional organoids. The processes related to clearance and synaptic pruning are often dysregulated in pathological conditions. For instance, excessive or aberrant microglia-mediated synaptic pruning has been implicated in AD, while impaired clearance of protein aggregates and cellular debris contributes to pathology in some neurodegenerative diseases (Dantzer, 2017; Heneka et al., 2024).

Finally, integrated organoid systems enable the modeling of feedback mechanisms between different types of cells that cannot be dynamically modeled in simpler models (Han et al., 2025; Sun et al., 2022). Changes in neuronal activity can influence vascular tone and immune signaling, while immune activation can alter both neuronal and vascular function. This bidirectional communication reflects the complexity of the in vivo brain environment and its central for the homeostasis of the tissue. The feedback loops are also very important in disease conditions, as seen in MS, where immune-mediated BBB disruption causes further immune cell infiltration, or in AD, where vascular dysfunction, chronic inflammation, and neurodegeneration affect each other in a self-amplifying cycle (Zierfuss et al., 2024).

## 7 Current challenges

3D neural models, including organoids and bioprinted constructs, have significantly advanced the study of brain development and neurodegenerative diseases. However, despite these advances, current systems remain limited in their ability to fully recapitulate the structural, functional, and pathological complexity of the human brain. Importantly, the primary limitation of these models is not the absence of biological components, but the lack of precise control over the microenvironmental parameters that govern cellular behavior and disease progression.

A fundamental challenge lies in the developmental immaturity of in vitro neural systems. Most models are derived from induced pluripotent stem cells or neural stem cells and exhibit gene expression profiles resembling early developmental stages rather than aged brain tissue (Kelava and Lancaster, 2016). As a result, key processes associated with biological aging, which are central to late-onset neurodegenerative diseases such as AD and PD, are poorly represented. While strategies such as telomere shortening have been explored to induce aging-like phenotypes, their integration into 3D systems remains limited (Harley et al., 2023). In addition, long-term culture stability remains a bottleneck, as prolonged maintenance of neural constructs introduces cellular stress and disrupts maturation, restricting current models to short-term observations rather than progressive disease modeling (Giandomenico et al., 2020).

Beyond intrinsic biological limitations, the lack of vascularization and efficient transport remains a major constraint. In the absence of functional vasculature, diffusion limits oxygen and nutrient delivery, leading to hypoxia, metabolic stress, and the formation of necrotic cores. This results in heterogeneous tissue maturation, where viable outer layers coexist with dysfunctional inner regions (Grebenyuk and Ranga, 2019). Although microfluidic systems and perfusable vascular networks have begun to address these issues, replicating physiologically relevant flow, shear stress, and vascular–neural interactions remain a significant challenge (Grebenyuk et al., 2023).

Another critical limitation is the incomplete representation of cellular diversity and neuroimmune complexity. Key components of the brain microenvironment, including microglia, oligodendrocytes, and vascular cells, are frequently absent or underrepresented. Even when present, these cell types often lack functional maturity or appropriate interactions. This is particularly limiting for modeling neuroinflammation, myelination, and BBB function, all of which play central roles in neurodegenerative disease progression (Grenier et al., 2019).

At the tissue level, functional connectivity and regional specificity remain difficult to achieve. In vivo, neural circuits rely on long-range axonal projections and coordinated interactions between distinct brain regions. These features are largely absent in conventional organoid systems, limiting the ability to model network-level dysfunction. While assembloid approaches partially address this limitation by combining region-specific tissues, they still fall short of reproducing the full complexity of brain-wide connectivity (Grenier et al., 2019).

From a technical perspective, reproducibility and standardization present major barriers. Organoid systems exhibit significant variability due to self-organization, differences in differentiation protocols, and reliance on undefined matrices such as Matrigel. This results in inconsistencies in size, cellular composition, and maturation state, complicating data interpretation and scalability (Hofer and Lutolf, 2021; Sandoval et al., 2024). In contrast, bioprinting provides greater control over spatial organization, but introduces challenges related to biomaterials and process optimization.

A central engineering challenge in bioprinting lies in the design of bioinks. Effective bioinks must balance bioprintability, mechanical stability, and biocompatibility while supporting neuronal differentiation and network formation. High-viscosity materials improve structural fidelity but increase shear stress during bioprinting, reducing cell viability (Bilkic et al., 2022). Conversely, low-viscosity formulations favor cell survival but lack mechanical integrity. Crosslinking methods, particularly those involving UV light or reactive chemistries, can further introduce cytotoxic effects. While natural biomaterials provide bioactive environments and synthetic materials offer tunability, no current formulation fully satisfies the requirements for modeling the diverse and dynamic neural microenvironment (Boni et al., 2018).

Finally, scalability, cost, and technical complexity limit the translational potential of current platforms. Organoid production is labor-intensive and difficult to standardize for high-throughput applications, while bioprinting technologies require specialized equipment and controlled environments. The use of patient-derived stem cells adds additional variability and complexity. As a result, despite their biological relevance, current models remain constrained in their predictive power for clinical translation.

Collectively, these challenges highlight the need to transition from descriptive biological systems toward controllable engineering platforms. Future progress will depend on the integration of vascularization strategies, microfluidic systems, advanced biomaterials, and standardized fabrication methods. Rather than increasing complexity alone, next-generation models must enable precise control over spatial organization, cellular composition, and microenvironmental dynamics. The convergence of organoid technologies and bioprinting offers a promising path forward, combining biological self-organization with engineering precision to develop predictive, scalable, and clinically-relevant neural models.

## 8 Future directions

3D bioprinted neural microenvironments have rapidly emerged as transformative platforms for modeling neurodegenerative diseases, bridging the gap between reductionist in vitro systems and complex in vivo biology. Advances in biomaterials, stem cell technologies, and biofabrication have enabled the construction of neural tissues that capture key aspects of tissue architecture, ECM composition, and multi-cellular coordination (Choi et al., 2025; Papadimitriou et al., 2020; Li et al., 2025). Unlike conventional models, bioprinting enables precise spatial organization of cells and biomolecules, allowing controlled reconstruction of neuronal networks, synaptic connectivity, and axonal guidance, which are essential for faithfully modeling disease progression (Cadena et al., 2021; Choi et al., 2025; Shiwarski et al., 2021; Ayan et al., 2020). Unlike solely organoid systems that rely on stochastic self-organization, bioprinting provides deterministic control over cellular architecture and microenvironmental cues, thereby improving reproducibility and experimental tunability.

A major future direction lies in increasing microenvironmental complexity through multi-material and gradient bioprinting. Native neural tissues exhibit highly heterogeneous mechanical and biochemical landscapes that regulate differentiation, maturation, and function (Yang et al., 2021; Roth et al., 2021; Jain et al., 2020). Emerging bioprinting strategies, including multi-nozzle and microfluidic assisted systems, enable the generation of continuous gradients in stiffness, growth factors, and signaling molecules, thereby allowing more physiologically relevant modeling of disease specific niches (Huang et al., 2024). The development of tunable bioinks with defined rheological and biochemical properties will be critical for recapitulating region-specific neural environments.

Another important direction is the integration of organoid and bioprinting technologies. Organoid bioprinting combines the self-organizing capacity of stem cell derived tissues with the spatial precision of engineered systems, enabling the assembly of multi-cellular constructs with defined architecture and improved reproducibility (Huang et al., 2024; Li et al., 2025). This hybrid approach preserves developmental relevance while enabling controlled microenvironmental design. In this context, co-culture systems incorporating neurons, astrocytes, and microglia should be prioritized to capture non cell autonomous mechanisms of neurodegeneration (Zhang et al., 2025). Such degree of biomimicry, functionalization, precise architecture and cell diversity allows for investigation of intercellular interactions, disease progression and drug response both in terms of mechanical and immune aspects that goes beyond animal models with improved fidelity, thus providing improved platforms for rapid drug discovery and testing for specific diseases (Budharaju et al., 2025).

Functionalization of bioprinted constructs represents an additional frontier. Integration of electroactive materials, biosensor arrays, and responsive reporter systems enables real time monitoring of electrophysiological activity and network dynamics. These platforms provide the ability to correlate structural alterations with functional decline, which is essential for translational applications. Coupling such systems with high content imaging and omics level analyses will further enhance the understanding of neurodegenerative disease mechanisms and discovery of possible therapeutics (Khan and Ahmed, 2026).

Despite significant progress, standardization and scalability remain major challenges. Variability in bioink composition, bioprinting parameters, and validation strategies limits reproducibility and hinders translation toward high-throughput drug screening. The establishment of standardized characterization pipelines integrating mechanical, biochemical, and functional metrics will be essential for advancing these platforms toward industrial and clinical applications (Orr et al., 2025). In addition, challenges related to long term tissue stability, vascularization, and immune system integration remain critical barriers for modeling late-stage neurodegeneration.

The next phase of development will require the establishment of clear design parameters that link microenvironmental parameters to functional outcomes. Key variables such as spatial cell distribution, matrix stiffness, biochemical signaling gradients, and temporal maturation must be systematically integrated rather than optimized in isolation. Neuronal network stability depends not only on cell type composition but also on spatial organization and connectivity, while glial activation is highly sensitive to local mechanical and inflammatory cues. Establishing such design frameworks will enable the transition from descriptive models to programmable systems with improved predictive power (Li et al., 2025).

Looking forward, the convergence of microfluidics, organ-on-chip technologies, and 3D bioprinting is expected to drive the next generation of neural models. In parallel, artificial intelligence (AI) guided design of biomaterials, bioinks, and bioprinting architectures is emerging as a powerful strategy for optimizing construct performance (Fig. 6). Integration of these approaches with dynamic biofabrication concepts, including four-dimensional (4D) bioprinting and adaptive microenvironments, will enable the development of highly predictive and personalized disease models (Ramesh et al., 2024; Kim et al., 2020). Early work in AI-driven bioprinting already demonstrates the potential of data driven optimization in material design and process control, suggesting that future advances will increasingly rely on computationally guided biofabrication frameworks (Sun et al., 2023; Yu et al., 2025). In this regard computational and AI assisted approaches hold great promise in predictive bioink design both in research and clinical settings. Formulation of multi-component bioinks, balancing rheology, adjusting printability, presetting appropriate gelation rates, and predicting cell viability through machine learning and or other computational implementations promises elimination of months of empirical trial and error. “Digital twin” applications can further improve predictive capabilities by constructing digital temporal models of disease model constructs based on multi-modal raw data. In context of tissue engineering and disease modeling, processing of multi-modal raw data is an advantage provided explicitly by machine-learning implementations that fits perfectly into NMDC framework. Further, full automation of disease modeling, bioprinting and pertaining processes provides inherent opportunities for high throughput applications and scaling. Automated process promise multiplexing and tracking of disease models in arrays rather than one-on-one basis. However gaps between structural, physical and multi-cellular interactions need to be addressed in these future approaches.

**Fig. 6:**
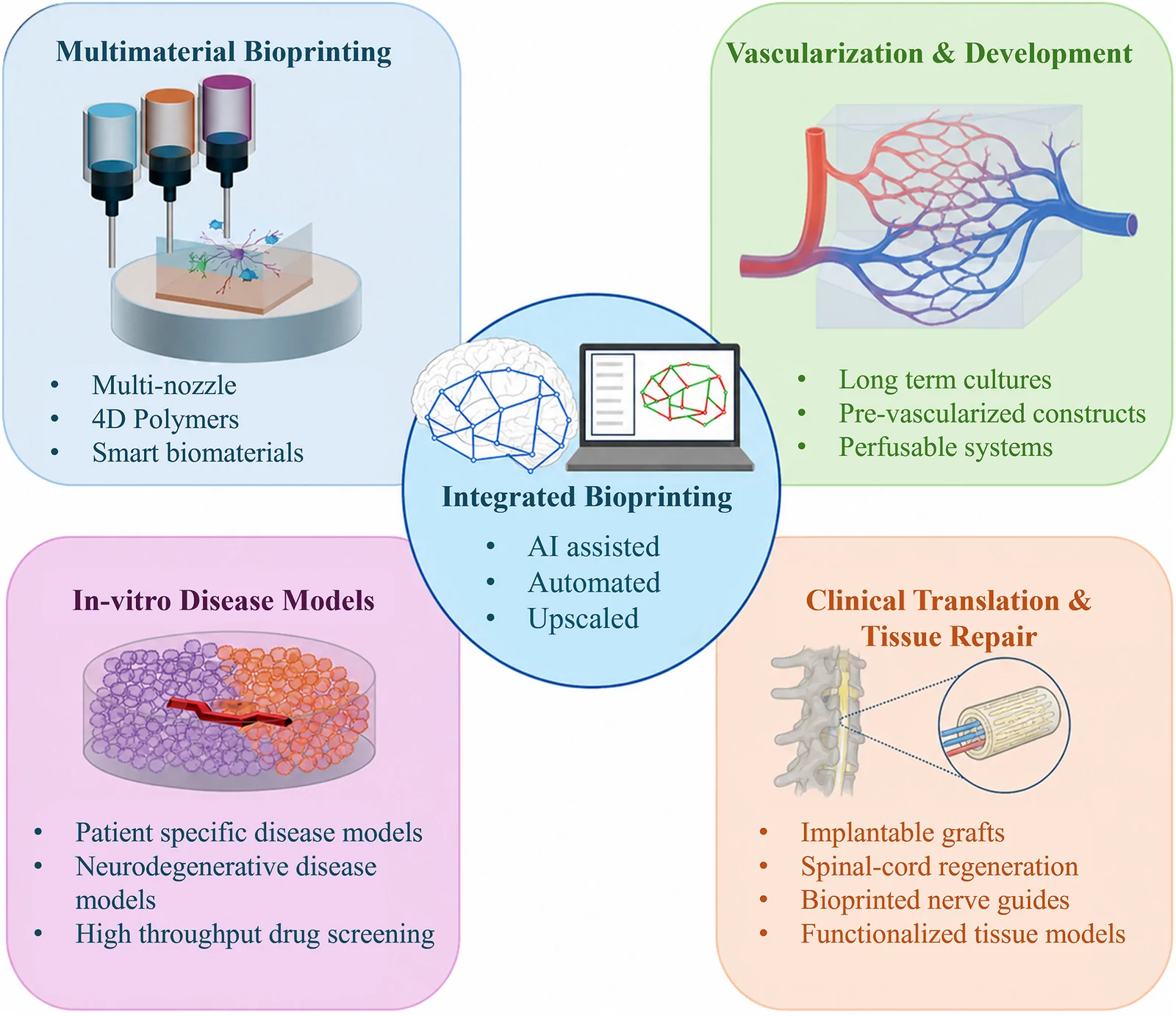
Future directions for integrated bioprinting strategies combining multi-material fabrication, vascularization and disease modeling to support high-throughput testing and accelerate clinical translation of next generation therapies.

3D bioprinted neural systems address this gap by enabling programmable control over cellular architecture and environmental cues. As such, these platforms hold strong potential to reduce the translational gap between preclinical studies and clinical outcomes by enabling patient specific disease modeling and predictive drug screening. Especially, the advent of 3D bioprinted constructs maintained on microfluidic platforms provides several promising aspects for streamlined high throughput drug screening and toxicology testing that does not rely on animal models and may even be comprised of patient homologous cells in order to maximize fidelity (Shukla et al., 2026).

Ultimately, progress in this field will depend on interdisciplinary integration across biomaterials engineering, stem cell biology, and computational design. This convergence will enable the development of controllable and biomimetic neural systems capable of advancing mechanistic discovery, therapeutic development, and personalized medicine in neurodegenerative diseases.

## CRediT authorship contribution statement

**Alp Deniz Yuzbasic:** Writing – original draft, Investigation, Conceptualization. **Begum Yagmur Yesil:** Writing – original draft, Visualization, Investigation, Conceptualization. **Damla Arslantunali Sahin:** Writing – review & editing, Supervision. **Bugra Ayan:** Writing – review & editing, Supervision, Methodology, Investigation, Conceptualization.

## Declaration of competing interest

The authors declare that they have no known competing financial interests or personal relationships that could have appeared to influence the work reported in this paper.

## Data Availability

No data was used for the research described in the article.
